# Transcriptome Assembly and Analysis of Tibetan Hulless Barley (*Hordeum vulgare* L. var. *nudum*) Developing Grains, with Emphasis on Quality Properties

**DOI:** 10.1371/journal.pone.0098144

**Published:** 2014-05-28

**Authors:** Xin Chen, Hai Long, Ping Gao, Guangbing Deng, Zhifen Pan, Junjun Liang, Yawei Tang, Nyima Tashi, Maoqun Yu

**Affiliations:** 1 Chengdu Institute of Biology, Chinese Academy of Sciences, Chengdu, Sichuan, China; 2 College of Life Sciences, Sichuan University, Chengdu, Sichuan, China; 3 University of Chinese Academy of Sciences, Beijing, China; 4 Tibet Academy of Agricultural and Animal Husbandry Sciences, Lhasa, Tibet, China; Agriculture and Agri-Food Canada, Canada

## Abstract

**Background:**

Hulless barley is attracting increasing attention due to its unique nutritional value and potential health benefits. However, the molecular biology of the barley grain development and nutrient storage are not well understood. Furthermore, the genetic potential of hulless barley has not been fully tapped for breeding.

**Methodology/Principal Findings:**

In the present study, we investigated the transcriptome features during hulless barley grain development. Using Illumina paired-end RNA-Sequencing, we generated two data sets of the developing grain transcriptomes from two hulless barley landraces. A total of 13.1 and 12.9 million paired-end reads with lengths of 90 bp were generated from the two varieties and were assembled to 48,863 and 45,788 unigenes, respectively. A combined dataset of 46,485 All-Unigenes were generated from two transcriptomes with an average length of 542 bp, and 36,278 among were annotated with gene descriptions, conserved protein domains or gene ontology terms. Furthermore, sequences and expression levels of genes related to the biosynthesis of storage reserve compounds (starch, protein, and β-glucan) were analyzed, and their temporal and spatial patterns were deduced from the transcriptome data of cultivated barley Morex.

**Conclusions/Significance:**

We established a sequences and functional annotation integrated database and examined the expression profiles of the developing grains of Tibetan hulless barley. The characterization of genes encoding storage proteins and enzymes of starch synthesis and (1–3;1–4)-β-D-glucan synthesis provided an overview of changes in gene expression associated with grain nutrition and health properties. Furthermore, the characterization of these genes provides a gene reservoir, which helps in quality improvement of hulless barley.

## Introduction

Barley (*Hordeum vulgare* L.) is among the most ancient cereal crops [1] and currently ranks fourth in terms of harvested area and tonnage of the world cereal production (http://faostat.fao.org). However, barley is the least utilized cereal for human food consumption and is usually cultivated either in regions unsuitable for wheat growing, or where barley is preferred for cultural reasons [2]. It was also neglected by plant breeders in Europe during the period of intensive crop improvement in the 20^th^ Century. However, it is currently gaining attention as a health food in Europe, North America and other non-traditional barley growing areas [3], [4]. Barley grains are rich in minerals; proteins and lysine and have a high β-glucan content, which inhibits cholesterol synthesis [5]–[7]. Hulless (naked) barley with caryopses that thresh free from the pales is preferred for human consumption [8]–[10]. Hulless barley also allows to omit a processing step, thus, providing an additional advantage for the food industry [11], [12]. Therefore, hulless barley is a potential resource for breeding new healthy food worldwide. The grain of barley is the major storage tissue. Different end uses require alternative quality characteristics of barley grain in terms of molecular composition of starch and proteins. So far, there has been limited research regarding metabolic profiling and gene expression patterns related to the metabolism of storage compounds during barley grain development.

The Qinghai-Tibet Plateau in western China has abundant hulless barley resources [13] and is considered as one of the main regions of domestication and diversity of cultivated barley [14], [15]. In the past millennia, people continuously modified local hulless barley populations to develop cultivars with increased grain yield. However, more efficient methods of barley production are needed to meet the increasing food demand imposed by climate change, potential food shortage, and demand for the use of grains as a renewable energy resource. The study of the genetic basis of agronomically important genes in hulless barley would certainly aid in developing better cultivation methods.

Genome sequencing is considered pivotal for solving key questions in crops and investigating the molecular mechanisms related to yield and quality. The International Barley Sequencing Consortium (IBSC) has made great achievements in the genomic sequencing of barley [16]. Meanwhile, numerous molecular technologies have also been applied to generate a greater functional understanding of barley, including microarrays [17]–[19], Affymetrix arrays [20], [21], cDNA-AFLP [22], SAGE [23], [24] and molecular markers [25]. These technologies have helped in generating data from more than 15 tissues or organs at various developmental stages and under diverse environmental conditions [17], [18]. However, the primary focus of these studies is usually on malting and feed characteristics. In this study, we conducted *de novo* transcriptome sequencing and analyses of the developing grains from two Tibetan hulless barley landraces, which have long been used as human food. A large number of unigenes were assembled, functionally annotated, and their expression accumulation was also calculated. We further analyzed the transcripts related to seed storage protein, starch, and β-glucan synthesis along with those identified in the Morex transcriptome data set [16]. This study provides abundant resources for identification of genes required for quality improvement in barley.

## Materials and Methods

### Ethics Statement

No specific permits were required for the described field studies as well as for the location where the experimental materials were planted. No endangered or protected species were involved in our field studies. The GPS coordinates of the three planting fields were 30°34′N, 103°53′E.

### Plant materials and RNA isolation

Two local varieties of Tibetan hulless barley, XQ754 and Nimubai (used and known as tribute barley), were conserved by the Tibet Academy of Agricultural and Animal Husbandry Sciences. Nimubai has a higher amylose content (33.9%) and β-glucan content (7.5%) as compared to XQ754, which had 27.2% amylose and 6.0% β-glucan (data collected from 2009–2010 in Chengdu). The hulless barley plants were cultivated in October, 2010 and grown under normal conditions in the three fields in Chengdu, Sichuan Province of China.

Grains of Nimubai and XQ754 plants were sampled at 5, 10, 15, 20, and 25 days after pollination (dap) for RNA extraction. Each sample consisted of grains from nine individuals. Total RNA was extracted from the grains using Trizol Reagent (Takara) and Fruit-mate for RNA purification (Takara), according to the manufacturer's instructions. The concentration and quality of RNA samples were determined using a Nano Drop 2000 micro-volume spectrophotometer (Thermo Scientific, Waltham, MA, USA). Equal amounts of RNA from each sample of the identical accessions were pooled to construct two cDNA libraries [26], [27].

### *De novo* transcriptome sequencing, assembly and evaluation

The library construction and sequencing were performed by the Beijing Genomics Institute (BGI)-Shenzhen, Shenzhen, China (http://www.genomics.cn). Briefly, beads with Oligo (dT) were used to isolate poly(A) mRNA from total RNA. Fragmentation buffer was added to breakdown mRNA into short fragments. Random hexamer-primers were added to the shortened fragments (∼200 bp), and first-strand cDNA was synthesized. The second-strand cDNA was synthesized using buffer, dNTPs, RNaseH and DNA polymerase I. Short fragments were purified with QiaQuick PCR extraction kit after resolution with agarose gel electrophoresis. Sequencing adapters were ligated to the cDNA strands and suitable fragments were selected for the PCR amplification as templates. After PCR amplification, the pair-end sequencing (90 bp in length) was carried out using Illumina HiSeq 2000.

Raw sequence data was generated by the Illumina pipeline and clean reads were generated by filtering out adaptor-only reads, reads containing more than 5% unknown nucleotides, and low-quality reads (reads containing more than 50% bases with Q-value ≤20). Only clean reads were used in the following analysis. The sequences from the Illumina sequencing were deposited in the NCBI Sequence Read Archive (Accession numbers: SRR1032035, SRR1032036, SRX375649 and SRX378862).

To reduce the data complexity, each library was assembled to unigenes separately with the program Trinity [28] using the follow parameters: group_pairs_distance = 250, path_reinforcement_distance  = 70, min_glue  = 2, min_kmer_cov  = 2 and other default parameters. After assembly by Trinity, all contigs from two samples were combined, and the redundancy of contigs was removed by the TGICL [29] and Phrap assemblers (http://www.phrap.org/) for obtaining distinct sequences (All-Unigenes). The following parameters were used to ensure quality of assembly: a minimum of 95% identity between contigs, a minimum of 35 overlapping bases, a minimum of 35 scores and a maximum of 20 unmatched overhanging bases at sequence ends.

In addition to the evaluation of the quality of the assemblies, the known 26,159 high-confidence genes [16] combined of RNA-seq-derived and barley flcDNAs-derived sequences were considered as references in this study, and were used to Blast against each assembly with Blastn (E-value <1e-10) [30]. Based on the Blast results, the averages of sensitivity and accuracy of each assembly were considered. Sensitivity or transcriptome coverage was determined as the ratio of the sum of all uniquely aligned segment lengths to the reference length. Accuracy was determined as the ratio of the sum of all unique aligned segment lengths to the assembled transcript lengths.

### Functional annotation and classification

Blastx alignment (E-value <1e-5) between unigenes and protein databases such as nr, Swiss-Prot, KEGG, COG and GO was performed, and the best-aligning results were used to determine the sequence direction and coding regions (CDS) and its amino acid sequence of unigenes. When different databases conflicted, the results were prioritized in the order: nr, Swiss-Prot, KEGG, GO and COG. When a unigene did not align to any of the databases, ESTScan [31] was used to decide its sequence direction and CDS.

A non-redundant unigene set “All-Unigenes” assembled from the two unigene sets were aligned by Blastx to protein databases (nr, Swiss-Prot, KEGG and COG) with E-value<1e-5, and proteins (including their protein functional annotations) having the highest sequence similarity with the given unigenes were retrieved. With nr annotation, the Blast2GO program [32] was used to get GO annotation of the All-Unigenes. WEGO software [33] using the GO functional classification for all All-Unigenes was used to understand the distribution of gene functions. The KEGG database (V56.0, Oct. 1, 2010) [34], [35] was employed to annotate the pathway of these unigenes.

### SNPs Identification

To detect the single nucleotide polymorphisms (SNPs) of XQ754 and Nimubai compared to the ESTs of barley (NCBI), 525,781 ESTs were downloaded from NCBI website (http://www.ncbi.nlm.nih.gov/). For the ESTs have high redundancy, clustering and assembly were performed by TGICL [29] and Phrap assemblers with the same parameters as mentioned previously, and a reference data set of 61,902 unigenes was generated. Thereafter, we realigned all the clean reads from each library onto the reference sequence separately using SOAP aligner with default parameters. SNPs were detected using SOAPsnp [36] with default parameters. To ensure the quality of SNP, we used the follow cutoff to filters: MinQual (minimal Quality form SOAPsnp) ≥20; Max_soap_rep <1.5; MinDist ≥5; MinDepth ≥5; MaxDepth <10000 [36], [37]. 29 SNPs in the CDS of eight genes encoding enzymes for starch and β-glucan synthesis were validated using Sanger sequencing.

### Differential Gene Expression Analysis

For gene expression analysis, the number of reads that uniquely aligned to a unigene was calculated and then normalized to RPKM (reads per kb per million reads) [38]. The RPKM method eliminates the influence of different gene lengths and sequencing levels on the calculation of gene expression. Therefore the calculated gene expression can be directly used for comparing the difference of in gene expression among samples. To identify differentially expressed genes between two samples, a statistical analysis of the frequency of each unique-match read in each library was performed by referring to “the significance of digital gene expression profiles” [39]. The P value was used to identify differentially expressed genes following the described formula [39], wherein N1 and N2 represent the total clean read numbers of unique-match reads in Samples 1 and 2, respectively, and gene A holds x and y unique-match reads in Samples 1 and 2, respectively.
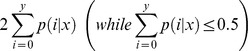



or
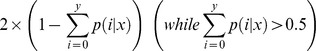





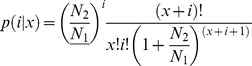



FDR (False Discovery Rate) was used in multiple hypothesis testing to correct for P value [40]. Following the formula below, assuming R differentially expressed genes had been selected, S genes of those were really differential expressed, whereas V genes indicated no difference which were false positive. The FDR value should not exceed 0.01, if the error ratio (Q = V/R) was required to be below a specified cutoff (0.01). FDR-values were calculated according to the previous algorithm [40].
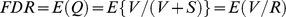



To judge the significance of gene expression differences, we used FDR ≤0.001, the Ratio ≥2 (the ratio of RPKM values). The genes with significant differential expression levels were subjected to GO function and KEGG pathway analyses.

### Q-PCR validation

The expression levels of ADP-glucose pyrophosphorylase small subunit gene, starch synthase IIa gene, 13s globulin gene and seven randomly selected genes were comfirmed using quantitative real-time PCR (Q-PCR). Q-PCR was performed using the same samples used for RNA-seq analysis. First-strand cDNA was synthesized using M-MLV reverse transcriptase (TaKaRa) according to the manufacturer's instructions. The cDNA was used as a template for Q-PCR. Unigenes and primers (designed using Primer Premier 5.0, Premier Biosoft International, Palo Alto, CA, U.S.) are listed in Table S1. The cDNA reaction mixture was diluted to five folds. The Q-PCR mixture (20 µl total volume) contained 10 µl of iQ SYBR green supermix (Bio-Rad), 0.5 µl of each primer (10 µM), 2 µl of cDNA, and 7 µl of RNase-free water. The reactions were performed on Chromo4 real-time PCR detector system (Bio-Rad, United States) according to the manufacturer's instructions. The Q-PCR program was performed after pre-incubation at 95°C for 5 min, followed by 40 cycles of denaturation at 95°C for 15 s, annealing at 60°C for 15 s, and extension at 72°C for 15 s. Template free controls for each primer pair were included in each run. The specificity of Q-PCR primers was confirmed by melting curve. The data were managed with the Gene Expression Analysis for iCycler iQ Real-Time PCR Detection System (Bio-Rad, Hercules, CA, USA) and normalized to that of the housekeeping gene EF (elongation factor 1α). The correlation coefficient (Pearson) of differential expression ratios between RNA-Seq and qRT-PCR was analyzed by using SPSS software 18.0 (http://www-01.ibm.com/software/analytics/spss/).

### Differentially expressed genes (DEGs) related to grain quality and expression pattern

Sequence similarity searches were performed using publicly available sequences from monocot species and *Arabidopsis* by Blastn (E-value <1e-10) to identify unigenes related to seed storage proteins and enzymes of starch and cellulose synthesis.

Patterns of gene expression in the germinating grain (4 day) embryos (EMB Embryo), roots (ROO) shoots from seedlings (LEA) (10 cm stage), early developing inflorescences (5 mm (INF 1) & 15 mm (INF 2)), developing tiller internodes (NOD) (six- leaf stage; sectioned between arrows), immature grains [5day post anthesis (dpa) (CAR5) & 15 dpa (CAR15)] were determined by RNA-seq in barley cv. Morex [16]. Representative transcript for one gene was chosen as those that had the maximum ORF extension. A transcript with the RPKM level above 0.4 was viewed as an expressed transcript.

## Results

### Transcriptome sequencing, *de novo* assembly, and quality evaluation

Sequencing of the XQ754 and Nimubai transcriptomes resulted in 13,069,860 and 12,918,520 clean reads, both with Q20 scores of 92.2% (Table 1). The GC contents of the two varieties were 56.5% and 56.2%, respectively. *De novo* assembly of XQ754 and Nimubai transcriptomes resulted in 48,863 and 45,788 unigenes with the average transcript length of 444 bp and 413 bp, respectively.

**Table 1:** Summary of *de novo* assemblies for two accessions.

Samples	Total Reads	Total Nucleotides (nt)	Unigenes	All-Unigenes
XQ754	13,069,860	1,176,287,400	48,863	46,485
Nimubai	12,918,520	1,162,666,800	45,788	

For the annotation, the two datasets were combined to form a non-redundant collection (All-Unigenes) containing 46,485 unigenes with an average length of 542 bp. About 62.0% (28,631) of the All-Unigenes were in the range of 300–500 bp; 11.8% (5,487) were longer than 1,000 bp, and no All-Unigene was shorter than 200 bp (Figure S1). Sequence similarity analysis was performed using the barley high-confidence gene set [16] to assess the assembly quality as queries for local Blast against the assembled unigenes. The average values of sensitivity and accuracy of the final assembly were 0.73 and 0.88, respectively, suggesting that the assembly was satisfactory.

### Characterization of the unigenes and CDS (coding sequences) prediction

**Figure 1:**
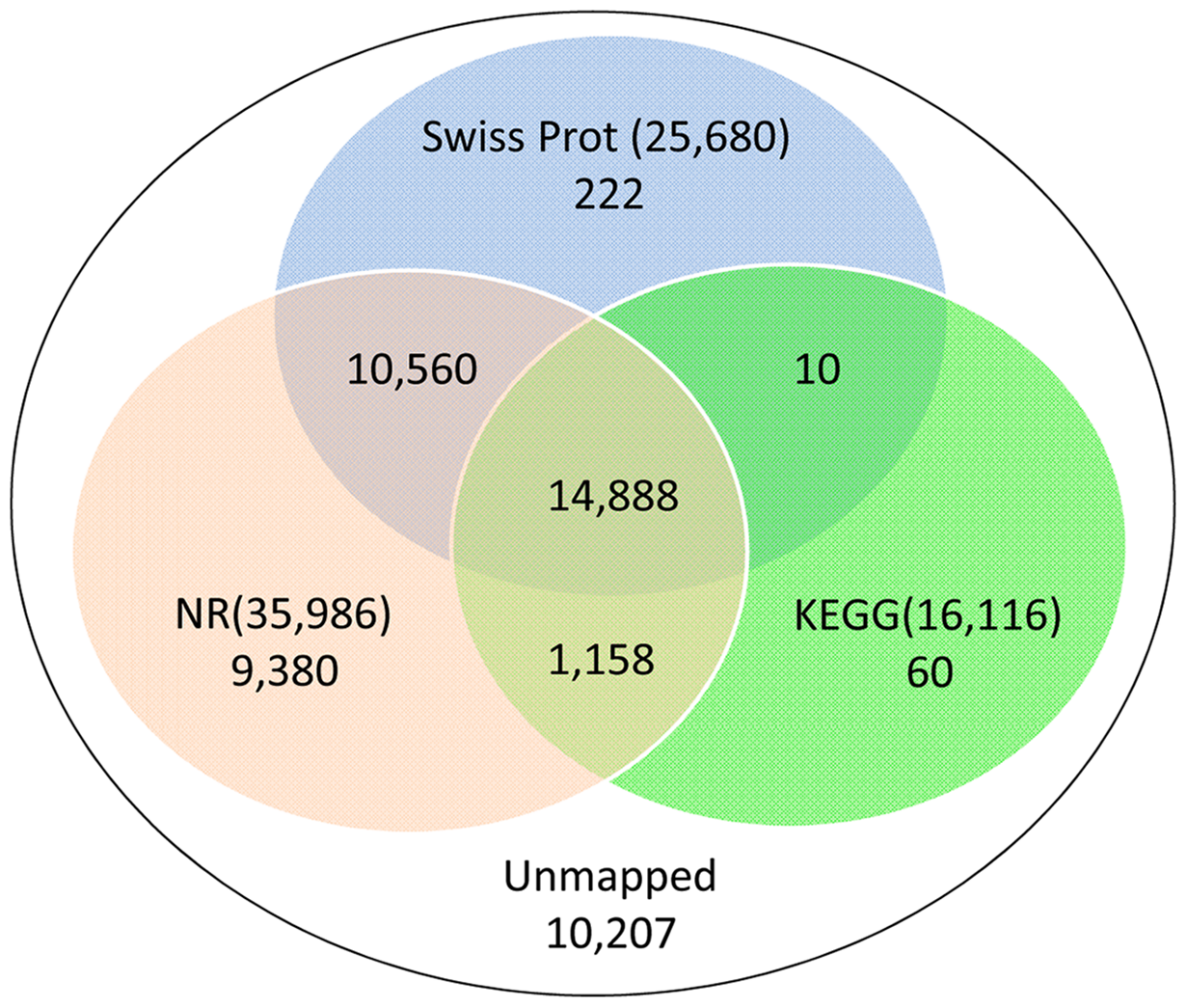
Distribution of Homologous genes in three public databases. The numbers of annotated and unmapped unigenes are indicated in the ellipses, respectively.

The All-Unigenes were aligned to three public protein databases (nr, Swiss-Prot and KEGG), and a total 36,278 unigenes were annotated, in which 35,986 (77.41%), 25,680 (55.24%) and 16,116 (34.67%) unigenes were annotated by nr, Swiss-Prot, and KEGG databases, respectively (Figure 1). The sequences direction of CDS (coding region sequences) and their amino acid sequences were acquired for among 38,229 unigenes, among which 36,307 (78.1%) unigenes were determined by Blastx (E-value <1e-5) against the public protein databases of nr, Swiss-Prot, KEGG and COG, and 1,922 (4.1%) were predicted by ESTScan [31].

### Functional classification

**Figure 2:**
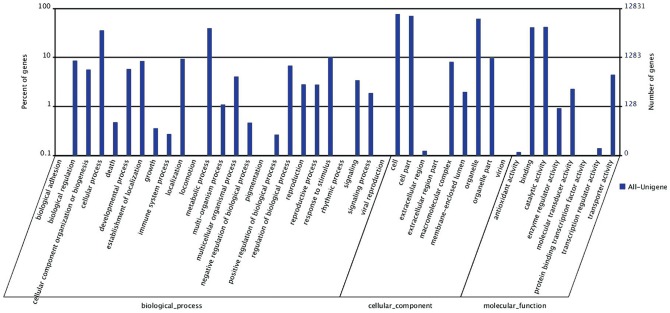
Go annotation of transcriptome. The x-axis indicates the categories and the y-axis indicates the number and proportion of All-Unigenes.

Among the 35,986 nr annotated All-Unigenes, only 12,831 could be further annotated with at least one GO term using Blast2GO [32], indicating that a large part of the nr annotation from hulless barley was not available for GO classifications. These 12,831 All-Unigenes were sorted in 42 GO terms (Figure 2), which were functionally assigned with the three GO terms as of Biological Process (19,010), Cellular Components (29,344) and Molecular Function (11,667). Within the biological process category, All-Unigenes were primarily assigned to GO terms of metabolic process (5,084 unigenes), cellular process (4,588 unigenes), response to stimulus (1,289 unigenes), biological regulation (1,105 unigenes) and establishment of localization (1,083 unigenes). With regard to the cellular component category, most All-Unigenes were assigned to cell (9,836 unigenes), cell part (9,066 unigenes), and organelle (7,879 unigenes). In the molecular function category, the major GO terms were catalytic activity (5,384 unigenes) and binding (5,263 unigenes). A similar profile was found in seeds of oat [41].

Clusters of Orthologous Groups of proteins (COGs) were delineated by comparing protein sequences encoded in complete genomes, representing major phylogenetic lineages. Each COG consisted of individual proteins or orthologous groups from at least three lineages and thus corresponded to an ancient conserved domain. The All-Unigenes were compared to the COG database using the Blastx algorithm specifying E-values of less than 10^−5^. A total of 13,579 All-Unigenes were annotated with 1,398 functional annotations in the COG database, which could be grouped into 25 functional categories belonging to cellular structure, molecular processing, biochemistry metabolism, signal transduction, etc. (Figure S2). Most All-Unigenes were assigned to general function prediction (4,256), followed by transcription (3,209), function unknown (3668), translation, ribosomal structure and biogenesis (3,207), posttranslational modification, protein turnover and chaperones (2,530), signal transduction mechanisms (1,096, 10.8%), cell wall/membrane/envelope biogenesis (2,381), replication, recombination and repair (2,340), cell cycle control, cell division and chromosome partitioning (2,248). Furthermore, 6,612 unigenes which might affect the quality of the grains were also identified. These unigenes were assigned to carbohydrate transport and metabolism; amino acid transport and metabolism; lipid transport and metabolism; energy production and conversion; and secondary metabolites biosynthesis, transport and catabolism.

We further analyzed biochemical pathways represented by the collection of unigenes. Using the KEGG database, which categorizes gene functions with emphasis on biochemical pathways, a total of 120 pathways represented by 16,116 All-Unigenes were predicted. These pathways in the developing grain of hulless barley have significant roles in biochemical for compound bio-synthesis, assimilation, degradation, and utilization and pathways involved in generation of precursor metabolites and energy. Plant metabolites are crucial for both plant life and human nutrition. Furthermore, these metabolites are important for enzymes involved in all steps in the major plant metabolic pathways including the Calvin cycle, TCA cycle, glycolysis, gluconeogenesis and the pentose phosphate pathway represented by unigenes derived from the hulless barley grain dataset. The functional significance of secondary metabolites in reproductive plant parts, particularly seeds of plants in natural ecosystems, is not well known. However, our study highlighted the unigenes associated with these parts, which can enhance our understanding of these metabolites. Furthermore, several unigenes involved in other important secondary metabolite biosynthesis pathways were found. These included the flavonoid biosynthesis pathway, which plays important roles in a number of biological processes and confers health-promoting effects against chronic diseases, such as cardiovascular diseases. Unigenes associated with carotenoid biosynthesis, which is indispensable to plants and plays a critical role in human nutrition and health were also found. Moreover, unigenes involved in several signaling pathways including ethylene pathway, programmed cell death (PCD), and abscisic acid (ABA)-mediated maturation were also found.

### Gene expression patterns

On the basis of RPKM, five expression patterns on relative expression levels were classified for 46,485 All-Unigenes. Pattern 1 contains eight unigenes in XQ754 and 14 unigenes in Nimubai with dramatically high RPKM values of 10,000 and 27,000, respectively. Pattern 2 consists of seven unigenes in XQ754 and five unigenes in Nimubai with very high RPKM value from 5,000 to 10,000. The two patterns include the barley stripe mosaic virus genes, resistance genes, hordein genes and a probable cytochrome P450 monooxygenase gene. There are 115 unigenes in XQ754 and 135 unigenes in Nimubai with high RPKM values (pattern 3) from 1,000 to 5,000. Some of these 115 unigenes are involved in grain development, response to stimulus, ribosome biogenesis, metabolic process, cation binding and gene expression (data not shown). There are 1,618 unigenes in XQ754 and 1,514 unigenes in Nimubai with RPKM value from 100 to 1,000 (pattern 4) and more than 80% unigenes of the two accessions have the RPKM value below 100 (pattern 5), and genes of these two patterns mainly function in grain development and nutrition biosynthesis. Over all, the pathways with most abundant transcripts according to the RPKM value are metabolic pathways, spliceosome, ribosome, plant-pathogen interaction, endocytosis, starch and sucrose metabolism and protein processing in the endoplasmic reticulum.

**Figure 3:**
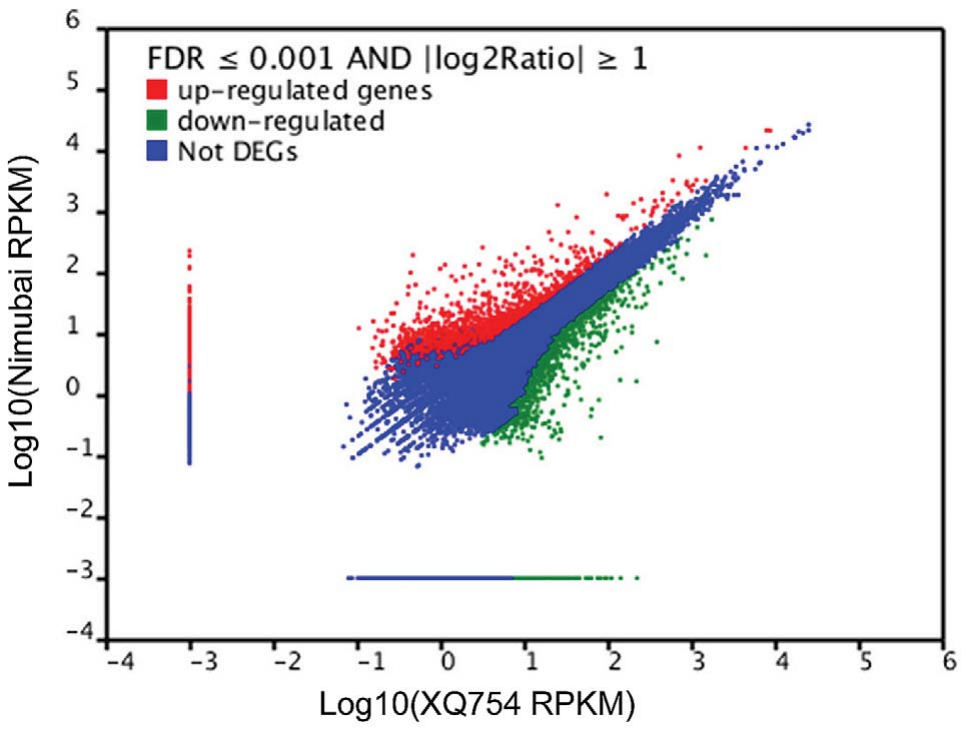
Gene expression levels of XQ754 and Nimubai. The differentially expressed genes are shown in red and green. Genes without expression changes are shown in blue. FDR ≤0.001 and ratio larger than 2.

We also compared the expression patterns of the two accessions and found 4,532 (9.7%) differently expressed unigenes. Of this, 1,381 unigenes were expressed at higher levels and 3,151 unigenes were expressed at comparatively lower levels in Nimubai as compared to those in XQ754 (Figure 3). The GO analysis of the differentially expressed unigenes revealed that within the biological process category (Figure S3), differential expressed unigenes were primarily assigned to GO terms of metabolic process (574 unigenes), cellular process (480 unigenes), response to stimulus (153 unigenes), biological regulation (118 unigenes) and localization (117 unigenes). In the cellular component category, most differentially expressed unigenes were assigned to cell (1,031 unigenes), cell part (937unigenes) and organelle (802 unigenes). In the molecular function category, the major GO terms were binding (553 unigenes) and catalytic activity (553 unigenes).

According to the annotations of nr, Swiss-Prot, KEGG, COG and GO, data mining of genes related to barley grain quality was performed. Altogether, 373 quality related transcripts belonging to starch metabolism (starch biosynthesis or degradation), grain storage protein synthesis (hordeins, globulins and glutelin), essential amino acids biosynthesis and degradation (asparagine, aspartate, lysine, methionine, and threonine), seed maturation, and seed development were identified (Table S2). We analyzed the expression levels of these unigenes in the developing grains of the two landraces and found that most of the unigenes showed little or no change in expression. Only 44 (11.8%) unigenes showed differences in expression, wherein 11 unigenes were expressed at higher levels, and 33 unigenes were expressed at comparatively lower levels in Nimubai than those in XQ754. In the two accessions, differentially expressed genes were mainly involved in biosynthesis and degradation of the aspartate family amino acids and starch metabolism. Furthermore, a remarkable expression of enzymes involved in methionine metabolism revealed the availability of sulfur-containing amino acids for protein synthesis during grain development. This is significant in designing strategies for modifying the nutritional value of barley seeds. Further research is needed to explain the specific functions of these genes on barley grain quality.

### Genes involved in starch biosynthesis

**Figure 4:**
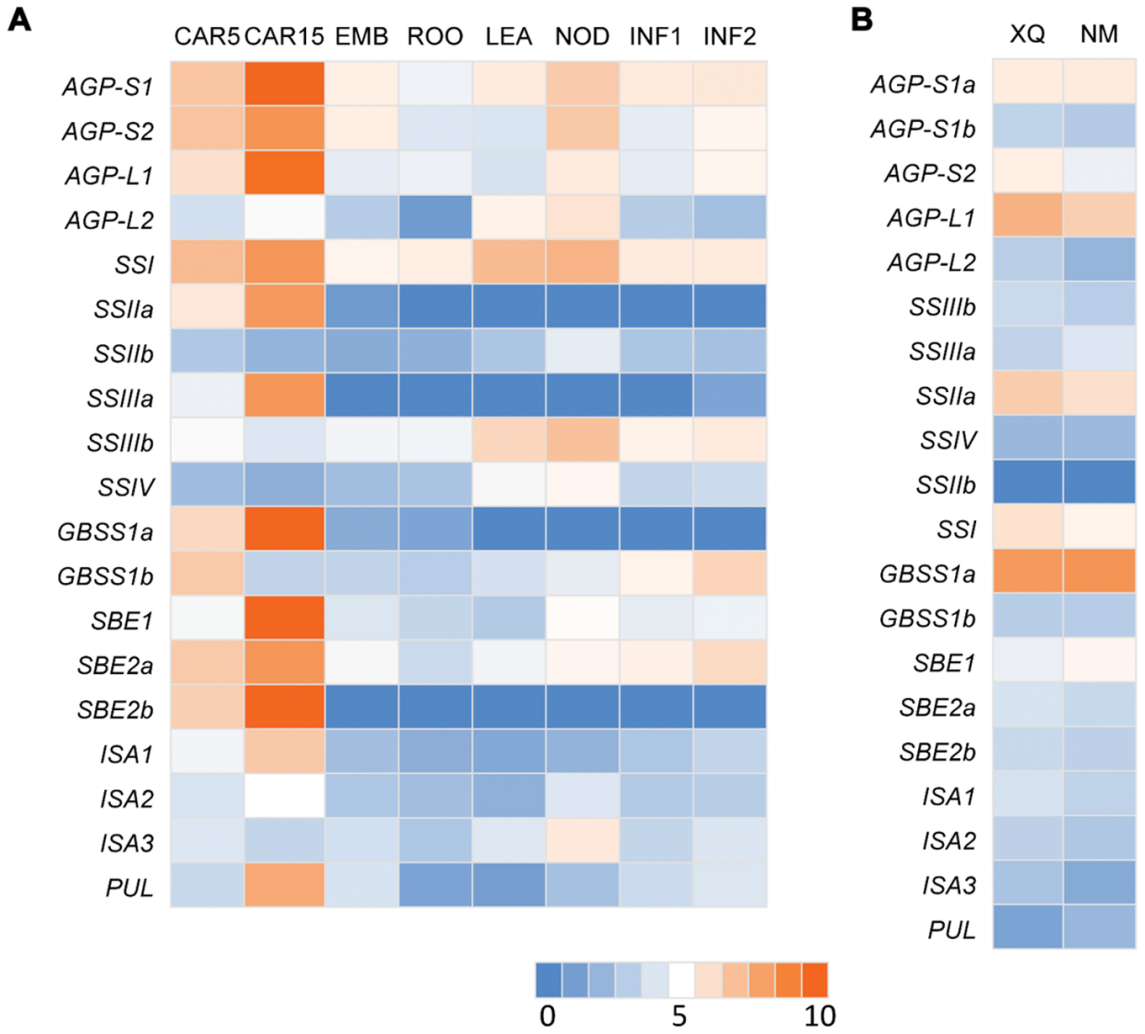
Heat map showing expression profiles of genes involved in starch biosynthesis. A) Gene expression profiles in eight tissues of Morex. B) Gene expression profiles of XQ (XQ754) and NM (Nimubai). Red color shows high expression level, while blue marks low expression level.

We further studied the transcripts involved in the synthesis of main storage nutrient in hulless barley grain. Starch comprises 70% of the dry weight of cereal seeds and provides up to 80% of the calories consumed by humans. Starch biosynthesis in the barley grains requires the coordinated activities of several core enzymes [42]–[47]. The All-Unigenes dataset and the transcriptome dataset of barley cultivar Morex [16] were searched by Blastn (E-value <1e-10) using the known enzyme sequences of Arabidopsis, maize, and rice as query. A total of 19 All-Unigenes relevant to starch biosynthesis enzymes were detected, including ADP-glucose pyrophosphorylase (AGPase), granule-bound starch synthase (GBSS), soluble starch synthase (SS), starch branching enzyme (SBE), starch debranching enzyme (DBE), isoamylase (ISA) and the pullanase (or beta-limit dextrinase; PUL) (Figure 4).

The AGPase, a heterotetrameric enzyme composed of two small (AGP-S) and two large (AGP-L) subunits, catalyzes the first key regulatory step in the starch biosynthetic pathways in all higher plants. Transcripts of *AGP-S1*, *AGP-S2*, *AGP-L1* and *AGP-L2* were detected in the two accessions and in all tested tissues of Morex (Figure 4). The *AGPS1* apparently encodes the transcripts for AGPS1a and AGPS1b, which differ only in their first exons. *AGP-S1a* and *AGP-S2* were abundantly expressed in the starchy grains of the two accessions, whereas *AGP-S1b* was found to be present only at a moderate level in the grain. *AGP-L1* had expression above 80 RPKM, while *AGP-L2* had expression below 10 RPKM in the developing grains of both XQ754 and Nimubai. Peak expression of *AGP-S1*, *AGP-S2* and *AGP-L1* was attained in 15 dpa grain (CAR15) and all AGPase transcripts except *AGP-L2* were strongly up-regulated at the grain filling stage (Figure 4).

Of the two currently known *GBSS* isoforms in barley, *GBSSIa* had a much higher expression level (>30 times) than *GBSSIb* in Nimubai and XQ754 grains. However, there were no significant differences between the two accessions. Furthermore, Morex data revealed that *GBSSIa* was mainly expressed in storage tissues and strongly up-regulated in 15 dap grain, whereas *GBSSIb* were not detected in grain but were found in transitory starch accumulated tissues, especially in INF1 and INF2 (Figure 4).

The transcriptome database screen also identified the unigenes of *SSI*, *SSIIa*, *SSIIb*, *SSIIIa*, *SSIIIb*, and a fraction of *SSIV* (Figure 4). In Morex, the gene expression of *SSIIIa* and *SSIIa* was restricted to grains compared with *SSI*, *SSIIb*, *SSIIIb* and *SSIV* which were also expressed in other tissues. In addition, the transcripts of *SSIIb*, *SSIIIb* and *SSIV* had an accumulation peak in the node but were expressed at relatively low levels during grain developing. *SSI* and *SSIIa* had the highest RPKM values as compared to the others in the two accessions accounting for more than 70% of the total SS expression. However, *SSI*, *SSIIa* in 5 dpa grain and *SSI*, *SSIIa and SSIIIa* in 15 dpa grain of Morex had the highest RPKM than other SSs (Figure 4). Nevertheless, the differentially expressed transcripts were not found among these SS enzymes between the two accessions.

Sequences of the corresponding transcripts of three *SBE*, three different *ISA* and the *PUL* were recovered. *SBE1* was expressed at remarkably high levels in 15 dap grains but was expressed at low levels in other tissues. A moderate level of *SBE2a* expression was found in all tissues but this expression peaked at 15 dap in the grain. *SBE2b* transcripts were only detected in the developing barley grains with the highest expressed level in 15 dap grain (Figure 4). *ISA1* transcripts were abundant in 15 dap grain and had low expression level in other tissues while *ISA3* transcripts were abundant in node and early grain. *ISA2* was barely expressed in all tissues involved; the *PUL* gene was highly expressed in 15 dap grain but had low expression levels in other tissues (Figure 4). Moreover, the expression levels of these unigenes did not show a notable difference between the two accessions.

### Genes related to β-glucan synthesis

**Figure 5:**
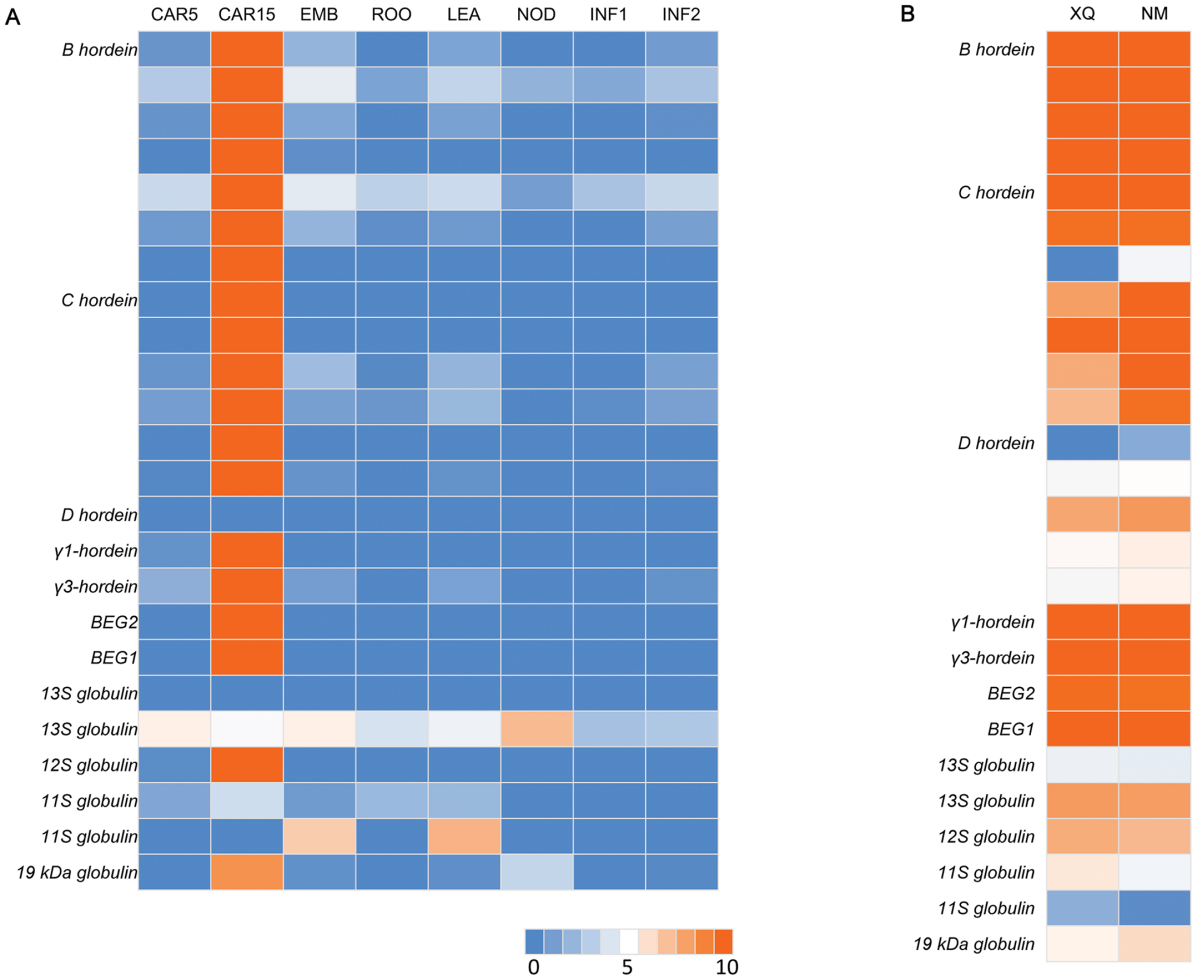
Heat map showing expression profiles of genes encoding cereal grain storage proteins. A) Gene expression profiles in eight tissues of Morex. B) Gene expression profiles of XQ (XQ754) and NM (Nimubai). Red color shows high expression level, while blue marks low expression level.

The β-glucans can significantly reduce the risk of serious human diseases such as type II diabetes, cardiovascular disease and colorectal cancer. Barley grain is particularly high in β-glucans and has a claimed usage in health products in more developed countries [16]. Two members of cellulose synthase-like (CSL) super family, *CslF* and *CslH*, have proved implication in β-glucan biosynthesis [48], [49]. In Morex, eight transcripts with close sequence similarity to known genes of *CslF* and *CslH* family [48], [49] were found, while a new transcript showed 64% identity to *CslF4* and another new transcript showed 70% identity to *CslF9* were also found. The two new transcripts were designated as *CslF4-like* and *CslF9-like* respectively (Figure 5). *CslF6* showed highest expression levels in all tissues tested, while *CslF9* showed second highest expression levels in grains. The expression of *CslF8* and *CslH1* were barely detected in immature grains but were high in roots and nodes, which is consistent to previous results obtained by quantitative PCR [50]. Meanwhile, *CslF3*, *CslF4*, *CslF7* and *CslF10* were not expressed in developing grains. In our investigation, four *Csl* genes, *CslF6, CslF8*, *CslF9,* and *CslH1* were detected in the two hulless accessions (Figure 5). *CslF6* showed highest expression levels followed by *CslF9*. *CslF8* and *CslH1* showed very low expression levels. The expression levels of *CslF9* in XQ were higher than those in NM while vice versa in the expression levels of *CslF8* and *CslH1.*

### Genes encoding grain storage proteins

**Figure 6:**
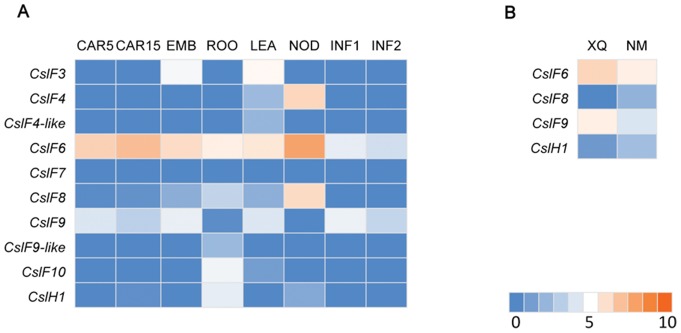
Heat map showing expression profiles of *HvCslF* and *HvCslH* gene families. A) Gene expression profiles in eight tissues of Morex. B) Gene expression profiles of XQ (XQ754) and NM (Nimubai). Red color shows high expression level, while blue marks low expression level.

Globulins are found in the embryo and outer aleurone layer of the endosperm. The structure and properties of the globulins are similar to the 7S vicilins of legumes [51]. Transcripts for eight *globulin* genes were found in XQ754, Nimubai, and Morex, including one *BEG1*, one *BEG2*, two *11S-like globulins*, one *12S-like globulin*, one*19kDa-like globulin* highly homologous to *19kDa globulin* gene of rice, and two transcripts with high homology to the *Setariaitalica 13S globulin* (Figure 6). The *BEG1* transcript shares 99% identity with previously reported barley embryo globulin gene which exhibits sequence similarity to 7S seed globulins of both monocots and dicots [52]. Distinct from *BEG1* (only 38% identity), a novel globulin transcript, temporarily designated as *BEG2*, was identified. *BEG2* was found to be homologous to the maize *GLB2*. Among the globulin genes, *BEG1* and *BEG2* were the most abundant transcripts followed by transcripts of a 13S-like and a 12S-like globulin in Nimubai and XQ754. *BEG1*, *BEG2* and the *12S-likeglobulin* transcript showed remarkably high accumulationin15 dap grain but were rarely expressed in 5 dap grain and other tested tissues of Morex. The *19kDa-like globulin* was expressed at comparatively lower levels in Nimubai, XQ754 and Morex but showed similar expression pattern as *BEG1*, *BEG2* and the *12S-like globulin* in Morex. One *11S globulin-like* transcript which was rarely expressed in the two accessions was not expressed in the grains of Morex, but showed high expression levels in embryo and leaf, while the other one lowly expressed in the two accessions showed low expression levels in all tested tissues of Morex. Furthermore, the expression of one *13S globulins-like* was ubiquitous in all tested tissues at a low level in Morex but at a comparatively high level in the grains of the two accessions. However, the transcript that was undetected in Morex showed a lower expressed in Nimubai and XQ754. With the exception of one *11S globulin-like*, there was no significant difference in the globulin transcript between the two accessions.

Hordein accounts for ∼50% of the total protein in the mature grains, and could be classified into four groups named B, C, D and γ-hordeins based on their electrophoretic mobilities [53]. In Nimubai and XQ754, four *B-hordeins*, seven *C-hordeins*, five *D-hordeins*, and two *γ-hordeins* transcripts were found and most of them were highly expressed. Morex shows different transcript numbers of B, C, and D types. Only one transcript of D-hordein was detected and its expression level is unavailable. The five D-hordein transcripts of the two accessions shared over 92% identity with the transcript of D-hordein of Morex and 86% identity with the wheat y-type high molecular weight glutenin subunit gene.

### Validation of RNA-Seq data

Ten differentially expressed genes were selected to demonstrate the RNA-seq results using QPCR (Table S1). The Q-PCR data showed the similar trends with RNA-Seq samples. Linear regression [y  = αx+ b, (y  =  Q-PCR value; x  =  RNA-seq value)] analysis showed a high correlation (R  = 0.8391), indicating that the gene expression differences observed in transcript abundance between the two samples were highly credible (Figure S4).

### SNPs identification

By comparing our data with the public expressed sequence data of barley, we roughly found 17,608 and 14,121 SNPs in 7,335 and 6,285 unigenes of Nimubai and XQ754, respectively. Among them, a total of 8,893 SNPs were shared by both accessions and 13,943 SNPs were found between two hulless barley landraces. Within the detected SNPs, the transitions were much more common than transversions (about 2:1). Meanwhile, a similar number of A/G and C/T transitions and four transversion types (A/T, A/C, G/T, and C/G) were detected. We identified 29 SNPs in the CDS of eight genes encoding enzymes for starch and β-glucan synthesis. Fourteen SNPs were found between the two accessions, in which 3 and 11 occurred in Nimubai and XQ754, respectively, and 15 SNP were shared by both accessions (Table 2). Nine SNPs (∼31% of total) were nonsynonymous and resulted in nine amino acid changes. All these 29 SNPs were validated in Nimubai, XQ754, and other 10 hulless barley landraces by Sanger sequencing (data not shown). Among these, 13 SNPs were also variable (Table 2) and the others are identical among all accessions of hulless barley tested.

**Table 2:** SNPs of genes involved in starch and β-glucan biosynthesis.

	Transcripts length	Coordinate	Ref. Nuc	SNP				AA alteration	
				XQ		NM			
				Nuc	depth(hit/total)	Nuc	depth(hit/total)		
*AGP-S1a*	1545	501	T	C*	24/24	C*	20/20		
*AGP-L1*	1572	360	T	C	99/101	C	47/64		
*SSIIb*	448	271	C	T	21/23	T	14/15		
		303	T	C	17/19	C	19/19		
		336	T	C	16/16	C	24/24		
*GBSS1a*	1829	529	T	C*	9/9	C*	14/14		
		789	A	G*	255/474	G*	255/593		
		795	T	C*	255/459	C*	255/540		
		837	A	G*	255/451	G*	255/538		
		1077	A	G*	104/104	G*	107/107		
		1272	C	A	239/239	A	255/267		
		1383	G	A*	128/138	A*	184/189		
*SBE1*	1811	711	A	G*	8/8	G*	17/17		
*CslF6*	2451	512	A	C	69/69	C	49/49	M-L	
*CslF9*	1829	1311	A	C	69/69	C	37/37	N-H	
*AGP-L1*	1572	519	A	G*	93/97	-			
*CslF6*	2451	439	T	C	14/14	-			
*CslF9*	1829	1682	G	A	8/8	-			
*SSIIIa*	1112	429	T	-		C	15/15	F-L	
		437	G	-		C	15/15		
		460	G	-		T	8/8	C-F	
		806	T	-		C	22/22		
		943	T	-		C	14/14	F-S	
*SSIIb*	448	421	C	-		G	10/10		
*GBSS1a*	1829	374	A	-		G*	11/11	I-V	
		423	G	-		A*	110/110	S-N	
		451	C	-		T*	138/140		
*SBE1*	1811	629	C	-		T*	11/11	S-L	
*CslF9*	1829	1815	T	-		G	14/14	W-G	

Note: Nuc, nucleotide; AA, amino acid; *, SNPs confirmed using Sanger sequencing. – indicates that the nucleotide is identical with the reference.

## Discussion

Hulled cultivated barley has been used in the brewing industry worldwide, however, lesser attention was paid on the grain quality of the hulless barley, which is the staple food at some barren regions or highland. Hulless barley has gained significant attention in recent years because of its potential health benefits such as higher β-glucan content than the hulled barley. Comparing to a long growing history and rich diversity in the Qinghai-Tibet Plateau, very few hulless barley cultivars have been developed for the modern UK or European agricultural systems. Thus, exploitation of germplasm resources and revealing the formation mechanism of grain quality in hulless barley will aid in the development of better hulless cultivars with desirable dietary characteristics. Here, we used high-throughput deep sequencing technology to profile the grain transcriptome of two Tibetan hulless barley landraces Nimubai and XQ754. We assembled 48,863 and 45,788 unigenes in two samples and constructed a combined non-redundant data set of 46,485 All-Unigenes. A total of 36,278 All-Unigenes could be functionally annotated, and the CDS and directions of 38,229 All-Unigenes were predicted.

Using Blast search and functional annotation, new transcripts with homology to the genes previously reported in other species could be identified. For instance, six new globulin transcripts (*BEG2*, two *11S-like globulins*, two *13S-like globulins* and one *19kDa-like globulin*) were predicted in the All-unigene dataset and Morex, respectively. Furthermore, two new transcripts *CslF9-like* and *CslF4-like* were detected in Morex. The deduced amino acid sequences of these new transcripts were compared with other known sequences and domains from NCBI (Figure S5–S11). Most of these new transcripts were validated by highly homogenous ESTs (Table S3) from full-length cDNAs in barley [54], [55]. Although their functional roles need further verification, all novel transcripts will help us to study the storage proteins and β-glucans synthesis. They will also provide valuable insights for identifying new genes that influence the grain quality and seed development.

We attempted to characterize the sequences and transcript accumulation of grain quality related genes encoding the seed storage proteins and the enzymes involved in starch and β-glucan biosynthesis in grains. Nineteen unigenes relevant to starch biosynthetic enzymes were detected. Among them, *AGP-S1* and *AGP-L1* were mainly expressed in the developing grain at high levels, suggesting their importance at the first step of starch biosynthesis. Moreover, they possibly associate to form a heterotetrameric cytosolic AGPase, similar to AGP-S2b and AGP-L2 of rice [56]. The chain elongation of amylose and amylopectin are distinctively catalyzed by the starch granule-bound form of starch synthase (GBSS) and soluble form of starch synthase (SS), respectively. Of the two GBSS isoforms, *GBSSIb* functions in non-storage plant tissues in which transitory starch accumulates, while *GBSSIa* is confined to storage tissues and has a much higher expression level than *GBSSIb* in grains of Nimubai and XQ754. *GBSSIa* then acts as the main limiting enzyme in the endosperm amylose production. This result is consistent with previous research in barley, rice and wheat [42], [43], [57]. However, the expression levels of *GBSSIa* in Nimubai and XQ754 were not significantly different in our study.

Among the SSs, *SSIV* gene was expressed in diverse tissues and at relatively low levels during grain filling and similar expression profiles were found in a Morex and rice [57]. The *SSIV* mutants of Arabidopsis show a striking reduction in the number of starch granules but an increase in starch granule size, indicating that SSIV could be selectively involved in the priming of starch granule formation [58]. Furthermore, the *SSIV* gene may not play typical roles as other SSs in the elongation of amylopectin chains during starch biosynthesis in barley. *SSI* and *SSIIa* of the two accessions and *SSI*, *SSIIa*, *SSIIIa* of Morex had the highest expression level among SSs.

In rice endosperm, *SSI* and *SSIIIa* are the major SS enzymes and *SSI* activity is higher than that of *SSIIIa*, constituting about 70% of the SS activity [59], which is consistent with other data of wheat [60] and maize [61]. Contrastingly, *SSII* and *SSIII* account for the major SS activities in potato tubers [62] and pea embryos [63]. In barley, we found that SSI and SSIIIb act extensively in diverse tissues, whereas SSIIa and SSIIIa mainly function during seed development. This suggests that the expression level of *SSI, SSIIa* and *SSIIIa* may be divergent among species, and their coordinated action might play a critical role in the grain amylopectin chain biosynthesis.

Comprehensively, *AGP-S1*, *AGP-L1*, *GBSSIa*, *SSI*, *SSIIa, SSIIIa, SBE1, SBE2b, ISA1* and *PUL*, which are mainly expressed in barley grain may significantly affect the starch biosynthesis in barley endosperm. There were no differentially expressed transcripts relevant to starch biosynthesis enzymes (except *AGP-S2*) between XQ754 and Nimubai. In starch biosynthetic pathway, each enzyme plays a distinct role, but presumably functions as part of a complex network. In this synthesis network, genes controlling amylopectin and amylose synthesis possibly interact [64], [65]. Thus, even though there is no divergence among the expression levels of the associated unigenes, the two accessions might have a different percentage of amylose mediated by multiple genes. In rice, the association analysis with individual starch synthesis-related genes revealed that *Wx* (*GBSS*) and *SSII-3* mainly control amylose content. *Wx* is likely the major gene and *SSII-3* acts as a minor effector. Under the same Wx background, varieties with different allelic *SSII-3* states show diverse amylose content [66]. SSIIa of barley accounts for the majority of amylopectin polymer elongation activity [67] and is highly homologous to SSII-3 of rice. In our results, Nimubai, which contains higher amylose content, also showed a higher RPKM ratio of *GBSSIa* to *SSIIa* as compared to XQ754. The elongation reactions for the chains of amylose and amylopectin are distinctively catalyzed by GBSS and SSs, respectively, thus the ratio of expression levels of *GBSSIa* to *SSIIa* might influence the ratio of amylose to amylopectin in barley.

β-glucan is a major constituent of the endosperm cell wall in barley grains [68], [69]. High content of β-glucan in barley grains has a negative effect on malting and pearling processes but is desirable for barley used as human food. Our analysis indicated that transcripts for the *CslF6* were the most abundant in developing barley grains, indicting its key role in controlling β-glucan synthesis in endosperm, which was also supported by analysis in barley β-glucanless mutants [70] and RNAi inhibition of *CslF6* in wheat grains [71]. Transcripts of the *CslF9* peaked earlier than *CslF6* and the previous study also described that the *CslF9* gene was transcribed at a stage when cellularization of the endosperm was completed and starch deposition had commenced, but disappeared somewhere between 12 and 15 days post-pollination [72]. In this study, we found that the *CslF9* transcript was expressed at a higher level in XQ754 than that in Nimubai (higher β-glucan content). This result is consistent with the previous study that *CslF9* appeared to be much more abundant in the elite malting variety ‘Sloop’ (lower) than the hulless barley ‘Himalaya’ (higher) [72]. This result suggests that CslF9 might not be a determinant of the β-glucan content and its role in β-glucan synthesis needs further study. Consequently, *CslF6* gene appears to encode the major β-glucan synthase, because of being constitutively expressed at much higher levels than all the other *CslF* genes in all tested tissues of barley. Other *CslF* genes may function as modifier in different stages of development or different tissues and organs. The *CslH1* has a proven function in β-glucan synthesis in barley. In this study, *CslH1* exhibited low expression levels in both hulless landraces, as well as in Morex, which is consistent with previous report. However, we noted that it is expressed at significantly higher level (∼2.7-fold) in Nimubai than that in XQ754. These results imply that *CslH1* may affect the total accumulation of β-glucan in barley grains independent of *CslF6*.

Cereal seed proteins are a source of primary nutrition for humans and livestock and have a great influence on the utilization of the grains in food processing. They usually account for about 10–15% of the dry weight of the seed and are mainly composed of globulins and prolamins [73], [74]. Eight globulins related transcripts were identified that showed similar expression patterns in hulled and hulless barley with the exception of one *13S globulin*. The *BEG1* and *BEG2* and *12S-like globulin* transcripts were highly expressed in hulled and hulless barley grains specifically. They encode globulins containing two ‘Cupin’ domains as those in13S-like globulins. This is consistent with prior research that the accumulation of *Beg1* mRNA was noted beginning 15–20 dpa of the developing barley grain [75]. Thus BEG1, and BEG2 and 12S-like globulins appear to function solely as main storage globulins.

Prolamins are the major endosperm storage proteins in most cereal grains. The allelic variation observed in hordeins and its influence on the food making, and malting quality is noteworthy. The B-hordeins and C-hordeins, encoded by the *Hor2* loci and *Hor1* loci, consist of 20–30 genes per haploid of barley genome [76], [77]. However, the D-and γ-hordeins, encoded by the *Hor3* and *Hor5* loci [78], [79], have minor members and the extent of polymorphism is unclear. The transcript numbers of B-hordein, C-hordein, and D-hordein between hulless and hulled genotypes were diverse and showed high variability. One D-hordein transcript was found in Morex; the sequence analysis of a 120-kb D-hordein region reported one D-hordein in that region [80], whereas five expressed D-hordein transcripts were found in the two hulless barleys. It is not known whether the increased number of D-hordein transcripts is caused by diverse members in the two accessions or improper sequence assembling.

In this study, we roughly identified more than ten thousand SNPs in the two hulless barley landraces. Twenty-nine SNPs identified in eight starch and β-glucan synthesis related genes were confirmed to be valid, indicating the high accuracy of SNP identification by transcriptome data. Thus, compared to the large-scale genomic sequencing, the transcriptome sequencing serves as an economic way for diversity detection. Furthermore, originating from expressed genes, all these transcriptome derived SNP might have great potential in the function associated analysis in the future.

## Supporting Information

Figure S1 Length distribution of All-Unigenes. The x-axis indicates the sequence length of unigenes and the y-axis indicates the number of unigenes, and the numbers of unigenes with a certain length are indicated on the top of the rectangle bars.
(PDF)

Figure S2 COG function classification. The capital letters in x-axis indicate the COG categories as listed on the right of the histogram and the y-axis indicates the number of unigenes.
(PDF)

Figure S3 Go annotation of differential expression unigenes. The x-axis indicates the categories and the y-axis indicates the number and proportion of differentially expressed unigenes.
(PDF)

Figure S4 Coefficient analysis between expression ratios obtained from RNA-seq and Q-PCR data of two landraces. ** indicates a significant difference at p≤0.01.
(PDF)

Figure S5 Alignment of amino acid sequences of putative7S globulin from barley cultivar Morex and the two accessions. Domains are indicated by bars and labels below the Alignment.
(PDF)

Figure S6 Alignment of amino acid sequences of putative11S-1 globulin from barley cultivar Morex and the two accessions. Domains are indicated by bars and labels below the Alignment.
(PDF)

Figure S7 Alignment of amino acid sequences of putative11S-2 globulin from barley cultivar Morex and the two accessions. Domains are indicated by bars and labels below the Alignment.
(PDF)

Figure S8 Alignment of amino acid sequences of putative13S globulin from barley cultivar Morex and the two accessions. Domains are indicated by bars and labels below the Alignment.
(PDF)

Figure S9 Alignment of amino acid sequences of putative19KD globulin from barley cultivar Morex and the two accessions. Domains are indicated by bars and labels below the Alignment. AAI_SS: Alpha-Amylase Inhibitors (AAIs) and Seed Storage (SS)protein subfamily; composed of cereal-type AAIs and SS proteins.
(PDF)

Figure S10 Alignment of amino acid sequences of putative CslF4 and CslF4-like proteins of barley cultivar Morex and the two accessions. Domains are indicated by bars and labels below the Alignment. Glycosyltransferase family A (GT-A) includes diverse families of glycosyltransferaseswith a common GT-A type structural fold.
(PDF)

Figure S11 Alignment of amino acid sequences of putativeCslF9 and CslF9-like proteins from barley cultivar Morex and the two accessions. Domains are indicated by bars and labels below the alignment. Glycosyltransferase family A (GT-A) includes diverse families of glycosyltransferases with a common GT-A type structural fold.
(PDF)

Table S1 Validation of ten differentially expressed genes using Q-PCR validation. Note: NM, Nimubai; XQ, XQ754.
(DOCX)

Table S2 List of genes related to seed quality.
(XLSX)

Table S3 New transcripts validated by highly homogenous ESTs of nr database.
(DOCX)
