# Supplementary material for: Transcriptome Assembly and Analysis of Tibetan Hulless Barley (Hordeum vulgare L. var. nudum) Developing Grains, with Emphasis on Quality Properties
Source: PLoS One. 2014 May 28;9(5):e98144. doi: 10.1371/journal.pone.0098144 (PMC4037191; doi:10.1371/journal.pone.0098144)
Supplement: Figure S1 — Length distribution of All-Unigenes. The x-axis indicates the sequence length of unigenes and the y-axis indicates the number of unigenes, and the numbers of unigenes with a certain length are indicated on the top of the rectangle bars. (PDF) [file pone.0098144.s001.pdf]

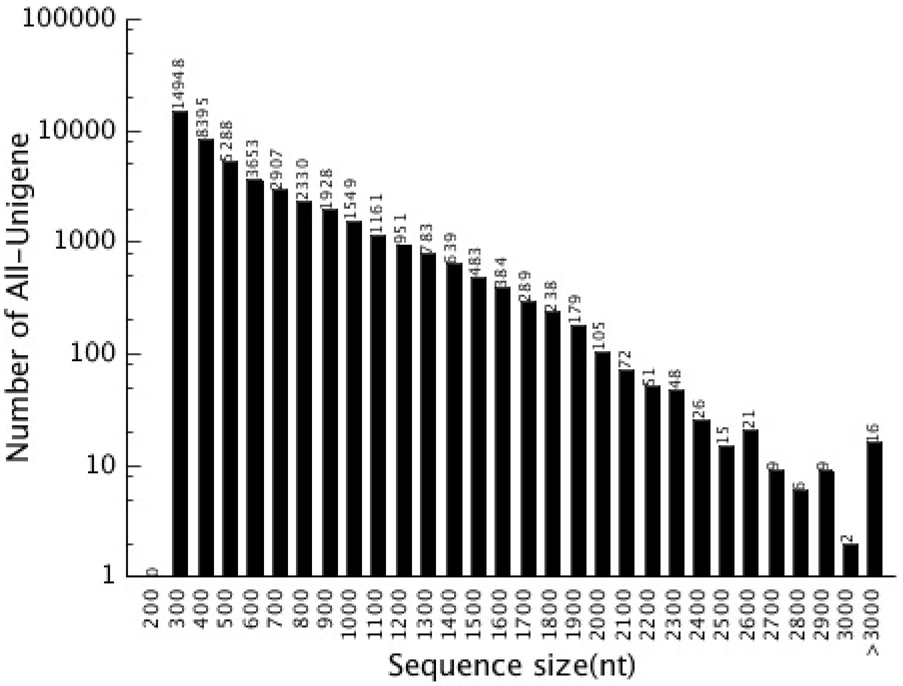

Figure S1 Length distribution of All-Unigenes. The x-axis indicates the sequence length of unigenes and the y-axis indicates the number of unigenes, and the numbers of unigenes with a certain length are indicated on the top of the rectangle bars.
