# Supplementary material for: Transcriptome Assembly and Analysis of Tibetan Hulless Barley (Hordeum vulgare L. var. nudum) Developing Grains, with Emphasis on Quality Properties
Source: PLoS One. 2014 May 28;9(5):e98144. doi: 10.1371/journal.pone.0098144 (PMC4037191; doi:10.1371/journal.pone.0098144)
Supplement: Figure S3 — Go annotation of differential expression unigenes. The x-axis indicates the categories and the y-axis indicates the number and proportion of differentially expressed unigenes. (PDF) [file pone.0098144.s003.pdf]

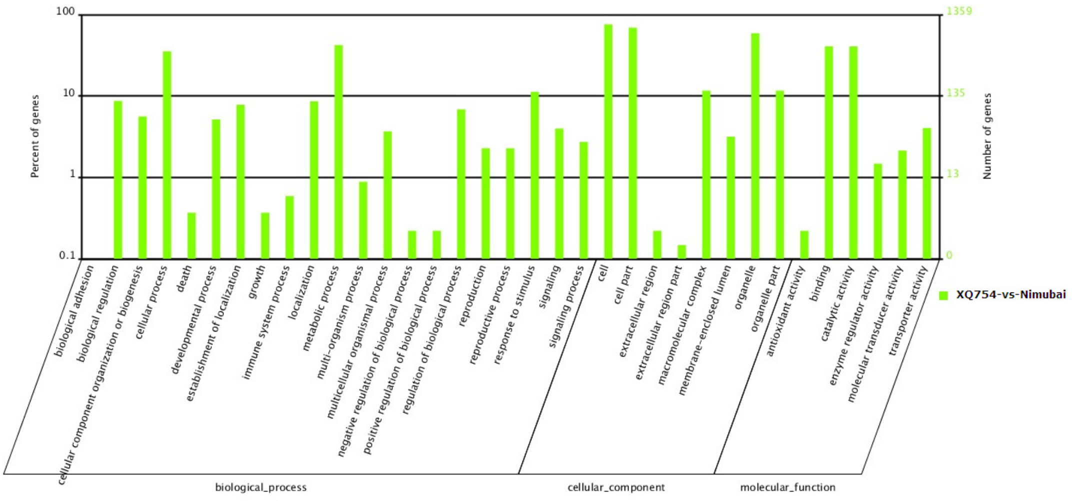

Figure S3 Go annotation of differential expression unigenes. The x-axis indicates the categories and the y-axis indicates the number and proportion of differentially expressed unigenes.
