# Supplementary material for: Transcriptome Assembly and Analysis of Tibetan Hulless Barley (Hordeum vulgare L. var. nudum) Developing Grains, with Emphasis on Quality Properties
Source: PLoS One. 2014 May 28;9(5):e98144. doi: 10.1371/journal.pone.0098144 (PMC4037191; doi:10.1371/journal.pone.0098144)
Supplement: Figure S4 — Coefficient analysis between expression ratios obtained from RNA-seq and Q-PCR data of two landraces. ** indicates a significant difference at p≤0.01. (PDF) [file pone.0098144.s004.pdf]

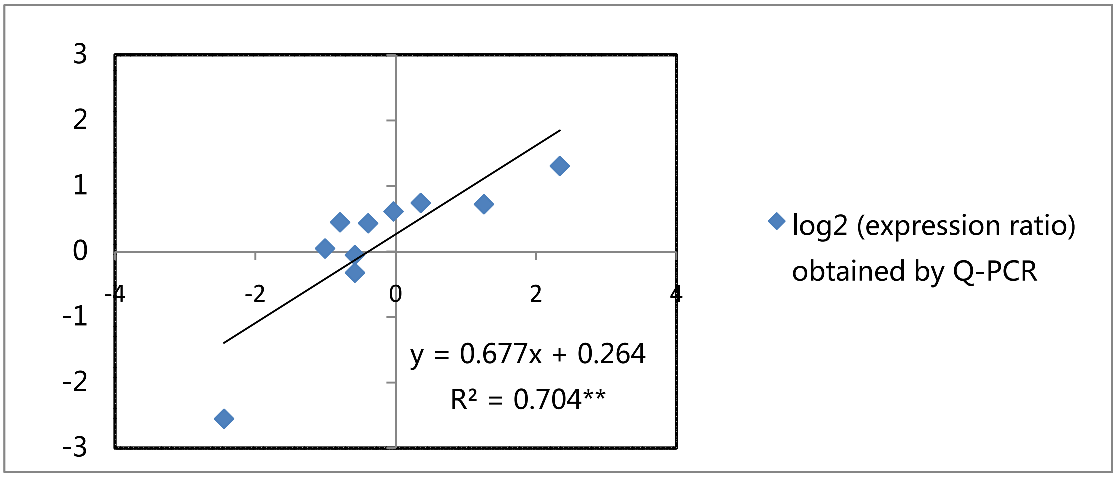

Figure S4 Coefficient analysis between expression ratios obtained from RNA-seq and Q-PCR data of two landraces. ** indicates a significant difference at $p \leq 0.01$.
