# Supplementary material for: Transcriptome Assembly and Analysis of Tibetan Hulless Barley (Hordeum vulgare L. var. nudum) Developing Grains, with Emphasis on Quality Properties
Source: PLoS One. 2014 May 28;9(5):e98144. doi: 10.1371/journal.pone.0098144 (PMC4037191; doi:10.1371/journal.pone.0098144)
Supplement: Figure S5 — Alignment of amino acid sequences of putative7S globulin from barley cultivar Morex and the two accessions. Domains are indicated by bars and labels below the Alignment. (PDF) [file pone.0098144.s005.pdf]

```
XP_004981475.1   ....................MGAMKRSPLVLLLLLSLLCSSLALASYSYGDAG..........GEGRSG.    39
Morex            MITNSSDQCTRQRAPTSITMKPAAARSPWLALAIVLSLCLSLSFASWDAEDEGRGSRRWQEGGDEGRSGE    70
Unigene          ......................................................................     0

XP_004981475.1   SGRPYHFGEERFRQWAQSRQGRFRVLERFTHELLEDAVGNYRVAELEAAPRAFLQPSHYDADEVMFVKEG   109
Morex            SGRPYHFGEESYREWAKSRHGHFKVLKRFDHELIRGSIGDYRVACLDAAPRAFLQPSHYDADEIAYVREG   140
Unigene          ...................................................AFLQPSHYDADEIAYVREG    19
                                              Cupin_1

XP_004981475.1   EGVVALLARGKRESFCVKEGDVLVIPAGAVVYSANTHGSKWFRVVMLLNPVSTKGRFEEFFPIG..GES.   176
Morex            EGVVVLLRNGKRESLCVREGDVFVIPAGSIVYSANTHRSKWFRVVMLLNPVSTPGSFQEFFPIGYGGEQQ   210
Unigene          EGVVVLLRNGKRESLCVREGDVFVIPAGSIVYSANTHRSKWFRVVMLLNPVSTPGSFQEFFPIGYGGEQQ    89
                                            Cupin_1

XP_004981475.1   PESFFSVFSDDVLQAAFNTRR.EEWEQVFEKQSKGE..ITTASEEQIRELSRSCSRTGRSHGGG......   237
Morex            PESFFSVFSDEVIRAAFNTRRREDVDRVFKRNSRGEGEISEASEEQIRELSRSCSRGGRGGGGGGGGGSG   280
Unigene          PESFFSVFSDEVIRAAFNTRRREDVDKVFKRNSRGEGEISEASEEQIRELSRSCSRGGRGGGGG...GSG   156
                   Cupin_1                                                 Cupin_1

XP_004981475.1   ESMWDIKLCSLTSKRPLHSNNHGRHYEITGDDCPQLRALDIEVGLTNLSRGSMTAPSYSTHADKLFVVLD   307
Morex            SEKEDIRPRSLTGEKPRYSNKHGRLHQITGDQCPHLRNLDLDVNLVNITRGSMTALRYTTRATKIVVVVE   350
Unigene          SDKEDIRPRSLTGEKPRYSNKHGRLHQITGDQCPHLRNLDLDVNLVNITRGSMTALRYTTRATKIVVVVE   226
                                            Cupin_1

XP_004981475.1   GSG...YFEMACPHLSSGRSSSPRRERGHGSREWGKEEEEEAEQEGGQKSRGYKQVKSRIREGSVIVIPA   374
Morex            AGNDGNYFEMACPHLSSS.GRSERRERGR.SEEQGGQEEEKGHGGEQEKSRGYRQVRAEIKVGSVIVLPA   418
Unigene          AGNDGNYFEMACPHLSSS.GRSERRERGRRSEEQGGQEEEQGHGGEQEKSRGYRQGRAEIKVGSVIVLPA   295
                                            Cupin_1

XP_004981475.1   GHPMTLVAGEDNNLAVLYFSVNARHDEKVFLVGSNGLLRQMDEAAKALAF.GAEKEKVDRVIGAQSDAVF   443
Morex            GHPATFVAGNEGNLAL......................................................   434
Unigene          GHPATFVAGNEGNLALLSFGVGANNDEEVFVTGGNSALKQLDEAAKALAFPQQVRELADKVIRAQTEAVF   365
                           Cupin_1

XP_004981475.1   LRGPNSRRVSSA.                                                            455
Morex            .............                                                            434
Unigene          HDGPQQQRRAFFD                                                            378
```

Figure S5 Alignment of amino acid sequences of putative7S globulin from barley cultivar Morex and the two accessions. Domains are indicated by bars and labels below the Alignment.
