# Supplementary material for: Transcriptome Assembly and Analysis of Tibetan Hulless Barley (Hordeum vulgare L. var. nudum) Developing Grains, with Emphasis on Quality Properties
Source: PLoS One. 2014 May 28;9(5):e98144. doi: 10.1371/journal.pone.0098144 (PMC4037191; doi:10.1371/journal.pone.0098144)
Supplement: Figure S6 — Alignment of amino acid sequences of putative11S-1 globulin from barley cultivar Morex and the two accessions. Domains are indicated by bars and labels below the Alignment. (PDF) [file pone.0098144.s006.pdf]

```
BAJ96050.1   MSASAKGGKALVKSDAGAYVAWSGTDQPELAAEGLGCGLLLLRPLSFALPHYADSPKFGY    60
Morex        MSASAKGGKALVKSDAGAYVAWSGTDQPELAAEGLGCGLLLLRPLSFALPHYADSPKFGY    60
Unigene      MSASAKGGKALVKSDAGAYVAWSGTDQPELAAEGLGCGLLLLRPLSFALPHYADSPKFGY    60
                                                                 Cupin_2

BAJ96050.1   VLRGSGVAGVLPVATEDASAAARERVVRLEAGDVIAVRTGDVSWWYNDSDGDTDDLSILF   120
Morex        VLRGSGVAGVLPVATEDASAAARERVVRLEAGDVIAVRTGDVSWWYNDSDGDTDDLSILF   120
Unigene      VLRGSGVAGVLPVATEDASAAARERVVRLEAGDVIAVRTGDVSWWYNDSDGDTDDLSILF   120
                                     Cupin_2

BAJ96050.1   LGDTERAVSPGDISYFFLAGGNSVLGGFDAGLLAGAWSGVTKEQAAAVFRSQPAVLLAGL   180
Morex        LGDTERAVSPGDISYFFLAGGNSVLGGFDAGLLAGAWSGVTKEQAAAVFRSQPAVLLAGL   180
Unigene      LGDTERAVSPGDISYFFLAGGNSVLGGFDAGLLAGAWSGVTKEQAAAVFRSQPAVLLAGL   180
                   Cupin_2

BAJ96050.1   STKLTGVCPREHDRKGLVVNAGQVAAGTLKAVTAADLAALGDLGISAVIGKLDPGAAQAP   240
Morex        STKLTGVCPREHDRKGLVVNAGQVAAGTLKAVTAADLAALGDLGISAVIGKLDPGAAQAP   240
Unigene      STKLTGVCPREHDRKGLVVNAGQVAAGTLKAGTAADLAARGGLGISAVIGKLDPGAAQAP   240
                                                  Cupin_1

BAJ96050.1   WVLREGAAQAVYVARGSARVQVSSSVGGETLLLDEEAAAGSVLVLPRFAVALISAGADGA   300
Morex        WVLREGAAQAVYVARGSARVQVSSSVGGETLLLDEEAAAGSVLVLPRFAVALISAGADGA   300
Unigene      WVLREGAAQAVYVARGSARVQVSSSVGGETLLLDEEAAAGSVLVLPRFAVALISAGADGA   300
                                     Cupin_1

BAJ96050.1   EWVSLIKSARPEVEQLTGDGSVLDGLTAQVVQASLSVAPELVELLGGRSAEPS          353
Morex        EWVSLIKSARPEVEQLTGDGSVLDGLTAQVVQASLSVAPELVELLGGRSAEPS          353
Unigene      EWVSLIKSARPEVEQLTGDGSVLDG............................          325
                              Cupin_1
```

Figure S6 Alignment of amino acid sequencesof putative11S-1 globulin from barley cultivar Morex and the two accessions. Domains are indicated by bars and labels below the Alignment.
