# Supplementary material for: Transcriptome Assembly and Analysis of Tibetan Hulless Barley (Hordeum vulgare L. var. nudum) Developing Grains, with Emphasis on Quality Properties
Source: PLoS One. 2014 May 28;9(5):e98144. doi: 10.1371/journal.pone.0098144 (PMC4037191; doi:10.1371/journal.pone.0098144)
Supplement: Figure S7 — Alignment of amino acid sequences of putative11S-2 globulin from barley cultivar Morex and the two accessions. Domains are indicated by bars and labels below the Alignment. (PDF) [file pone.0098144.s007.pdf]

```
AK2448848.1    MSAKAGKPLVQTDAGAYIAWSGADQPELATEGLGCGLLMLRPLGFALPHYADSNKFGYVL    60
Morex          MSAKAGKPLVQTDAGAYIAWSGADQPELATEGLGCGLLMLRPLGFALPHYADSNKFGYVL    60
Unigene        ...................WSGADQPELATEGLGCGLLMLRPLGFALPHYADSNKFGYVL    41
                                       Cupin_1

AK2448848.1    GGSGVAGVLPVAAKERVVRLEAGDVIAVRTGDVSWWYNDNDGHADDLSILFIGDTARALS   120
Morex          GGSGVAGVLPVAAKERVVRLEAGDVIAVRTGDVSWWYNDNDGHADDLSILFIGDTARALS   120
Unigene        GGSGVAGVLPVAAKERVVRLEAGDVIAVRTGDVSWWYNDNDGHADDLSILFIGDTA....    97
                                     Cupin_1

AK2448848.1    PGDISYFFLAGGNSVLSGLDPVLLTRAWPRVTEEQAATAFRSQPAVLLTRLSTKLAGVCP   180
Morex          PGDISYFFLAGGNSVLSGLDPVLLTRAWPRVTEEQAATAFRSQPAVLLTRLSTKLAGVCP   180
Unigene        ............................................................    97
                          Cupin_1

AK2448848.1    REDDRTGLVVNAGHVAAGTLKTVTASDLAGLGGLGFSAVLGRLEPGAARAPWVLREGAAQ   240
Morex          REDDRTGLVVNAGHVAAGTLKTVTASDLAGLGGLGFSAVLGRLEPGAARAPWVLREGAAQ   240
Unigene        ............................................................    97
                                           Cupin_1

AK2448848.1    AVYVARGSARVQVSAAAGGEKLLMDEEVAAGSLFVVPRFAVALVAAGAEGVEWVSLIKSA   300
Morex          AVYVARGSARVQVSAAAGGEKLLMDEEVAAGSLFVVPRFAVALVAAGAEGVEWVSLIKSA   300
Unigene        ............................................................    97
                                     Cupin_1

AK2448848.1    RPVVEHLTGEGSVFGGLTARVVQASLNVAPELVKL                            335
Morex          RPVVEHLTGEGSVFGGLTARVVQASLNVAPELVKL                            335
Unigene        ...................................                             97
                          Cupin_1
```

Figure S7 Alignment of amino acid sequences of putative11S-2 globulin from barley cultivar Morex and the two accessions. Domains are indicated by bars and labels below the Alignment.
