# Supplementary material for: Transcriptome Assembly and Analysis of Tibetan Hulless Barley (Hordeum vulgare L. var. nudum) Developing Grains, with Emphasis on Quality Properties
Source: PLoS One. 2014 May 28;9(5):e98144. doi: 10.1371/journal.pone.0098144 (PMC4037191; doi:10.1371/journal.pone.0098144)
Supplement: Figure S8 — Alignment of amino acid sequences of putative13S globulin from barley cultivar Morex and the two accessions. Domains are indicated by bars and labels below the Alignment. (PDF) [file pone.0098144.s008.pdf]

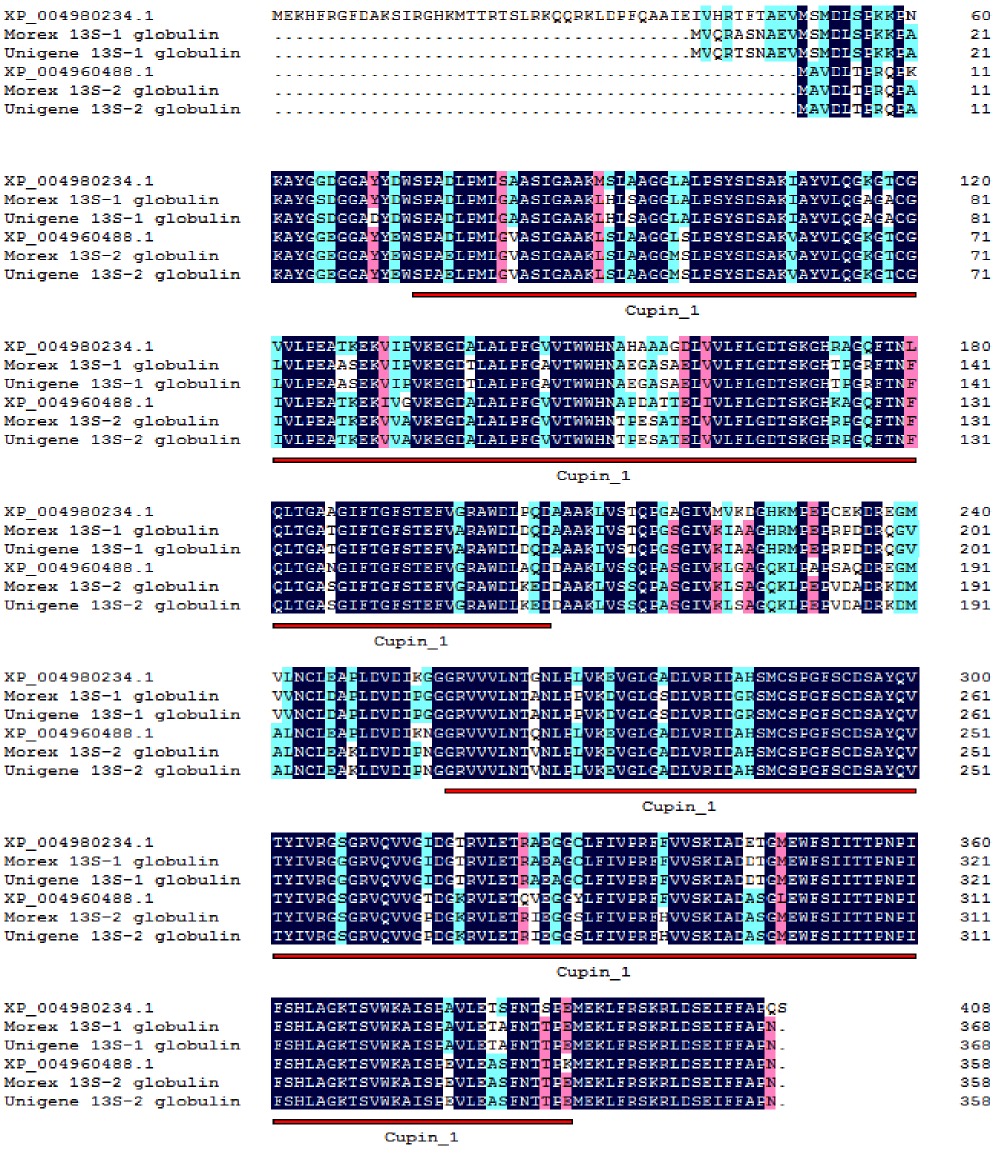

Figure S8 Alignment of amino acid sequences of putative13S globulin from barley cultivar Morex and the two accessions. Domains are indicated by bars and labels below the Alignment.
