# Supplementary material for: Transcriptome Assembly and Analysis of Tibetan Hulless Barley (Hordeum vulgare L. var. nudum) Developing Grains, with Emphasis on Quality Properties
Source: PLoS One. 2014 May 28;9(5):e98144. doi: 10.1371/journal.pone.0098144 (PMC4037191; doi:10.1371/journal.pone.0098144)
Supplement: Figure S9 — Alignment of amino acid sequences of putative19KD globulin from barley cultivar Morex and the two accessions. Domains are indicated by bars and labels below the Alignment. AAI_SS: Alpha-Amylase Inhibitors (AAIs) and Seed Storage (SS)protein subfamily; composed of cereal-type AAIs and SS proteins. (PDF) [file pone.0098144.s009.pdf]

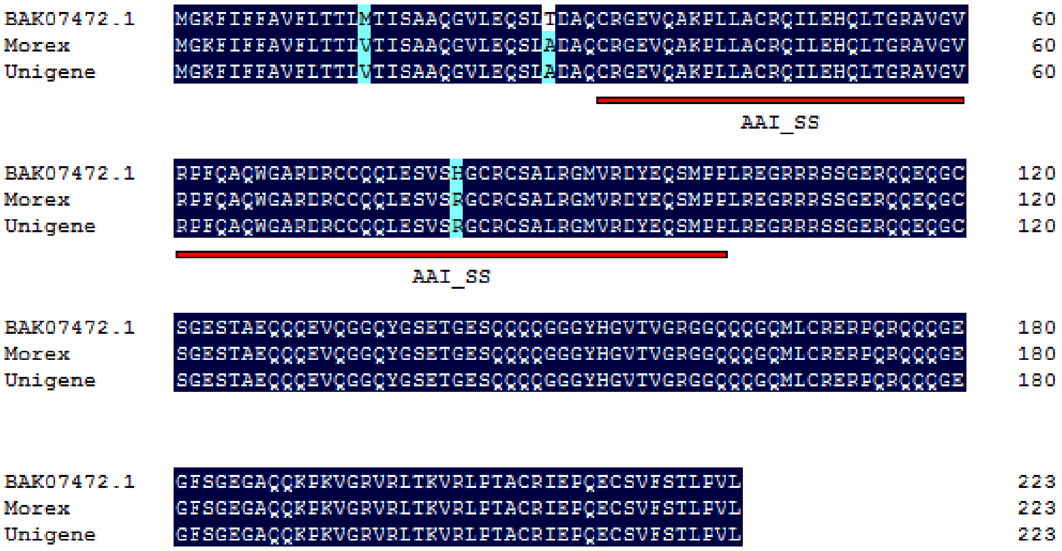

Figure S9 Alignment of amino acid sequences of putative19KD globulin from barley cultivar Morex and the two accessions. Domains are indicated by bars and labels below the Alignment. AAI_SS: Alpha-Amylase Inhibitors (AAIs) and Seed Storage (SS)protein subfamily; composed of cereal-type AAIs and SS proteins.
