# Supplementary material for: Transcriptome Assembly and Analysis of Tibetan Hulless Barley (Hordeum vulgare L. var. nudum) Developing Grains, with Emphasis on Quality Properties
Source: PLoS One. 2014 May 28;9(5):e98144. doi: 10.1371/journal.pone.0098144 (PMC4037191; doi:10.1371/journal.pone.0098144)
Supplement: Figure S10 — Alignment of amino acid sequences of putative CslF4 and CslF4-like proteins of barley cultivar Morex and the two accessions. Domains are indicated by bars and labels below the Alignment. Glycosyltransferase family A (GT-A) includes diverse families of glycosyltransferaseswith a common GT-A type structural fold. (PDF) [file pone.0098144.s010.pdf]

```
BAJ96916.1          MAPAVTRRANALRVEAPDGNAESGRASLAADSPAAKRAIDAKDDVWVAAAEGDASGASAGNGDRPPLFRTMKVKG    75
CslF4 Morex         MAPAVTRRANALRVEAPDGNAESGRASLAADSPAAKRAIDAKDDVWVAAAEGDASGASAGNGDRPPLFRTMKVKG    75
BAJ98864.1          .........MALRVEADAD..PKGRR.........ATDTDADKDVWVAAEEGDMSGASAG...RPLLFRTMKVKG    52
CslF4-like Morex    .........MALRVEADAD..PKGRR.........ATDTDADKDVWVAAEEGDMSGASAG...RPLLFRTMKVKG    52
                                                              Glyco_tranf_GTA_type

BAJ96916.1          SILHPYRFMILVRLVAVVAFFAWRLKHKNHDGMWLWATSMVADVWFGFSWLLNQLPKLNPIKRVPDLAALADQCG   150
CslF4 Morex         SILHPYRFMILVRLVAVVAFFAWRLKHKNHDGMWLWATSMVADVWFGFSWLLNQLPKLNPIKRVPDLAALADQCG   150
BAJ98864.1          SILHPYRFLILVRLVAIVAFFAWRVEHRNHDGMWLWATSMVADAWFGFSWLLNQLPKLNPTKRVPDLAALADRHD   127
CslF4-like Morex    SILHPYRFLILVRLVAIVAFFAWRVEHRNHDGMWLWATSMVADAWFGFSWLLNQLPKLNPTKRVPDLAALADRHD   127
                                              Glyco_tranf_GTA_type

BAJ96916.1          SSGDANLPGIDIFVTTVDPVDEPILYTVNTILSILATDYPVDKYACYLSDDGGTLVHYEAMIEVANFAVMWVPFC   225
CslF4 Morex         SSGDANLPGIDIFVTTVDPVDEPILYTVNTILSILATDYPVDKYACYLSDDGGTLVHYEAMIEVANFAVMWVPFC   225
BAJ98864.1          ...DAILPGIDVFVTTVDPVDEPVLYTVNTILSILAADYPVDKYACYLSDDGGTLVHYEAMLQVASFAALWVPFC   199
CslF4-like Morex    ...DAILPGIDVFVTTVDPVDEPVLYTVNTILSILAADYPVDKYACYLSDDGGTLVHYEAMLQVASFAALWVPFC   199
                                              Glyco_tranf_GTA_type

BAJ96916.1          RKHCVEPRSPENYFGMKTQPYVGSMAGEFMREHRRVRREYDEFKVRIDSLSTTIRQRSDAYNSSNKGDGVRATWM   300
CslF4 Morex         RKHCVEPRSPENYFGMKTQPYVGSMAGEFMREHRRVRREYDEFKVRIDSLSTTIRQRSDAYNSSNKGDGVRATWM   300
BAJ98864.1          RKHCIEPRSPENYFGMKTRPYVGGMAGEFMSDHRRVRREYGEFKVRIESLSTTIRRRSDAYNKGD..DGVHATWM   272
CslF4-like Morex    RKHCIEPRSPENYFGMKTRPYVGGMAGEFMSDHRRVRREYGEFKVRIESLSTTIRRRSDAYNKGD..DGVHATWM   272
                                              Glyco_tranf_GTA_type

BAJ96916.1          ADGTQWPGTWIEQVENHRRGQHAGIVQVILSHPSCKPQLGSPASTDNPLDFSNVDTRLPMLVYMSREKRPGYNHQ   375
CslF4 Morex         ADGTQWPGTWIEQVENHRRGQHAGIVQVILSHPSCKPQLGSPASTDNPLDFSNVDTRLPMLVYMSREKRPGYNHQ   375
BAJ98864.1          ADGTPWPGTWIEQADNHRRGQHAGIVEVMLDHPSCKPQLGFSASTDNPIDLSNVDTRLPMLVYISREKRSGYDNQ   347
CslF4-like Morex    ADGTPWPGTWIEQADNHRRGQHAGIVEVMLDHPSCKPQLGFSASTDNPIDLSNVDTRLPMLVYISREKRSGYDNQ   347
                                              Glyco_tranf_GTA_type

BAJ96916.1          KKAGAMNVMLRVSALLSNAPFVVNFDCDHYINNTQALRAPMCFMLDPRDGQNTAFVQFPQRFDDVDPTDRYANHN   450
CslF4 Morex         KKAGAMNVMLRVSALLSNAPFVVNFDCDHYINNTQALRAPMCFMLDPRDGQNTAFVQFPQRFDDVDPTDRYANHN   450
BAJ98864.1          KKAGAMNAMLRVSALLSNAPFVINFDCDHYINNSRALRAPMCFMLDPRDGQNTAFVQFPQRFDDVDPTDRYSNHN   422
CslF4-like Morex    KKAGAMNAMLRVSALLSNAPFVINFDCDHYINNSRALRAPMCFMLDPRDGQNTAFVQFPQRFDDVDPTDRYSNHN   422
                                              Glyco_tranf_GTA_type

BAJ96916.1          RVFFDGTMLSLNGLQGPSYLGTGTMFRRVTLYGMEPPRYRAENIKLVGKTYEFGSSTSFINSMPDGAIQERSITP   525
CslF4 Morex         RVFFDGTMLSLNGLQGPSYLGTGTMFRRVTLYGMEPPRYRAENIKLVGKTYEFGSSTSFINSMPDGAIQERSITP   525
BAJ98864.1          RVFFDGTMLSLNGLQGPTYLGTGTMFRRVALYGMEPPRYRAEDIKLVGKAVELGNSTPFLNSIPDGAIQERSITP   497
CslF4-like Morex    RVFFDGTMLSLNGLQGPTYLGTGTMFRRVALYGMEPPRYRAEDIKLVGKAVELGNSTPFLNSIPDGAIQERSITP   497
                                              Glyco_tranf_GTA_type

BAJ96916.1          VLVDEALSNDLATLMTCAYEDGTSWGRDVGWVYNIATEDVVTGFRMHRQGWRSMYCSMEPAAFRGTAPINLTERL   600
CslF4 Morex         VLVDEALSNDLATLMTCAYEDGTSWGRDVGWVYNIATEDVVTGFRMHRQGWRSMYCSMEPAAFRGTAPINLTERL   600
BAJ98864.1          VLVDDELNNDLATLMACGYEDGSSWGRDVGWVYNIATEDVVTGFRIHRQGWRSMYCSMEPAAFRGTAPINLTERL   572
CslF4-like Morex    VLVDDELNNDLATLMACGYEDGSSWGRDVGWVYNIATEDVVTGFRIHRQGWRSMYCSMEPAAFRGTAPINLTERL   572
                                              Glyco_tranf_GTA_type

BAJ96916.1          YQVLRWSGGSLEMFFSHSNALMAGRRIHPLQRVAYLNMSTYPIVTVFILAYNLFPVMWLFSEQFYIQRPFG.TYI   674
CslF4 Morex         YQVLRPP.......YPPSRCSSPTATLS..WPAAVSTLCSVSRTSTCRPTRSSRCSSWPTTSSPSCGSSPS.SST   665
BAJ98864.1          YQVLRWSGGSLEAFFSHSNALIASRRLHLLQRIAYLNMSIYPIATMFILAYSFFPVMWLFSEQSYYIQRPFGTFI   647
CslF4-like Morex    YQVLRWSGGSLEAFFSHSNALIASRRLHLLQRIAYLNMSIYPIATMFILAYSFFPVMWLFSEQSYYIQRPFGTFI   647
                                              Glyco_tranf_GTA_type

BAJ96916.1          MYLVGVIAMIHVIGMFEVKWAGITLLDWCRNEQFYMIGATGVYPTAVLYMALKLVTGKGIYFRLTSKQTDACSND   749
CslF4 Morex         SRGRSARTSCTSSAS............................................................   680
BAJ98864.1          MYLVAVIAMMHVIGMFEVKWAGITLLDWWRNEQFYMIAATGVYPTAVLYMALKLVRGKGIHFRLTSKQTGACSGE   722
CslF4-like Morex    MYLVAVIAMMHVIGMFEVKWAGITLLDWWRNEQFYMIAATGVYPTAVLYMALKLVRGKGIHFRLTSKQTGACSGE   722
                                     Glyco_tranf_GTA_type

BAJ96916.1          KFADLYTVRWVPLLFPTVAVLIVNVAAVGAAIGKAAAWGFFTDQARHVLLGMVFNVWILVLLYPFALGIMGKWGK   824
CslF4 Morex         ...........................................................................   680
BAJ98864.1          KFADLYAVRWVPLLIPTVAVLVVNVAAVGAAIGKAATWGFFTDQAWHAVLGMVFNVGTLVLLYPFALGIMGQWGK   797
CslF4-like Morex    KFADLYAVRWVPLLIPTVAVLVVNVAAVGAAIGKAATWGFFTDQAWHAVLGMVFNVGTLVLLYPFALGIMGQWGK   797

BAJ96916.1          RPIILFVMLIMAIGAVGLVYVAFHDPYPTDFSEVAASLGEASLTGPS                               871
CslF4 Morex         ...............................................                               680
BAJ98864.1          RPGILLVMLVMAIATVGLLYVALQQDGHSMSFLTRPSG.........                               835
CslF4-like Morex    RPGILLVMLVMAIATVGLLYVALQQDGHSMSFLTRPSG.........                               835
```

Figure S10 Alignment of amino acid sequences of putative CslF4 and CslF4-like proteins of barley cultivar Morex and the two accessions. Domains are indicated by bars and labels below the Alignment. Glycosyltransferase family A (GT-A) includes diverse families of glycosyltransferaseswith a common GT-A type structural fold.
