# Supplementary material for: Transcriptome Assembly and Analysis of Tibetan Hulless Barley (Hordeum vulgare L. var. nudum) Developing Grains, with Emphasis on Quality Properties
Source: PLoS One. 2014 May 28;9(5):e98144. doi: 10.1371/journal.pone.0098144 (PMC4037191; doi:10.1371/journal.pone.0098144)
Supplement: Figure S11 — Alignment of amino acid sequences of putativeCslF9 and CslF9-like proteins from barley cultivar Morex and the two accessions. Domains are indicated by bars and labels below the alignment. Glycosyltransferase family A (GT-A) includes diverse families of glycosyltransferases with a common GT-A type structural fold. (PDF) [file pone.0098144.s011.pdf]

```
ABZ01581.1          MASPAAVGGGRLADPLLAADVVVVGAKDKYWVPADEREILASQSSGGGEQDGRAPLLYRT   60
CslF9 Morex         MASPAAVGGGRLADPLLAADVVVVGAKDKYWVPADEREILASQSSGGGEQDGRAPLLYRT   60
CslF9_like Morex    ............................................................    0

ABZ01581.1          FRVKGFFINLYRLLTLVRVIVVILFFTWRMRHRDSDAMWLWWISVVGDLWFGVTWLLNQI   120
CslF9 Morex         FRVKGFFINLYRLLTLVRVIVVILFFTWRMRHRDSDAMWLWWISVVGDLWFGVTWLLNQI   120
CslF9_like Morex    ......................MVFFAWRVQHRDSDAMWLWWISVVGDFWFGLSWWLNQV   38
                                                                Glyco_tranf_GTA_type

ABZ01581.1          TKLKPRKCVPSISVLREQLDQPDGGSDLPLLDVFINTVDPVDEPMLYTMNSILSILATDY   180
CslF9 Morex         TKLKPRKCVPSISVLREQLDQPDGGSDLPLLDVFINTVDPVDEPMLYTMNSILSILATDY   180
CslF9_like Morex    PKLNPTICIPTISLLRQQFDLPDGGSNLPILDVFISTVDPVEEPMLHTMNSILSILATDY   98
                                        Glyco_tranf_GTA_type

ABZ01581.1          PVQKYATYFSDDGGSLVHYEGLLLTAEFAASWVPFCRKHCVEPRAPESYFWAKMRGEYAG   240
CslF9 Morex         PVQKYATYFSDDGGSLVHYEGLLLTAEFAASWVPFCRKHCVEPRAPESYFWAKMRGEYTG   240
CslF9_like Morex    PVDKYATYLSDDGGSLLHYDGLVETAKFAALWVPFCRKHGVEPRAPESYFGMKIR.PYTG   157
                                        Glyco_tranf_GTA_type

ABZ01581.1          SAAKEFLDDHRRMRAAYEEFKARLDGLSAVIEQRSEACNRAANEKEGCGNATWMADGSTQ   300
CslF9 Morex         SAAKEFLDDHRRMRAAYEEFKARLDGLSAVIEQRSEACNRAANEKEGCGNATWMADGSTQ   300
CslF9_like Morex    NMPEEFLDDHRRLRREYEEFRTRLDALFTVIPQRSEAHGRQDAKQGGGAKATWMADG.TQ   216
                                        Glyco_tranf_GTA_type

ABZ01581.1          WQGTWIKPAKGHRKGHHPAILQVMLDQPSKDPELGMAAS.SDHPLDFSAVDVRLPMLVYI   359
CslF9 Morex         WQGTWIKPAKGHRKGHHPAILQVMLDQPSKDPELGMAAS.SDHPLDFSAVDVRLPMLVYI   359
CslF9_like Morex    WPGTWTEPADGHRKGDHAGIVQVMLSQPSTEPQLGAPASPDDSPLDFSAVDVRLPMLVYV   276
                                        Glyco_tranf_GTA_type

ABZ01581.1          AREKRPGYDHQKKAGAMNVQLRVSALLSNAPFIINFDGDHYINNSQAFRAAMCFMLDPRD   419
CslF9 Morex         AREKRPGYDHQKKAGAMNVQLRVSALLSNAPFIINFDGDHYINNSQAFRAAMCFMLDPRD   419
CslF9_like Morex    SREKRPGYDHQKKAGALNVQLRVSALLSNAPFIINFDCDHYINNSQAFRAAMCFMIDRRD   336
                                        Glyco_tranf_GTA_type

ABZ01581.1          GADTAFVQFPQRFDDVDPTDRYCNHNRMFFDATLLGLNGIQGPSFVGTGCMFRRVALYSA   479
CslF9 Morex         GADTAFVQFPQRFDDVDPTDRYCNHNRMFFDATLLGLNGIQGPSFVGTGCMFRRVALYSA   479
CslF9_like Morex    GDNIAFVQFPQRFDDVDPTDRYANHNRMFFDATMLGMNGIQGPSYVGTGSMFRRVALYGA   396
                                        Glyco_tranf_GTA_type

ABZ01581.1          DPPRWRSDDAKEAKASHRPNMFGKSTSFINSMPAAANQERSVPSPATVGE...AELADAM   536
CslF9 Morex         DPPRWRSDDAKEAKASHRPNMFGKSTSFINSMPAAANQERSVPSPATVGE...AELADAM   536
CslF9_like Morex    DPPRWRPDDVKVLEN...PNKFGTSMTFINSIPVAANQERSVMSPVSLEEPATTEMADVM   453
                                        Glyco_tranf_GTA_type

ABZ01581.1          TCAYEDGTEWGNDVGWVYNIATEDVVTGFRLHRTGWRSTYCAMEPDAFRGTAPINLTERL   596
CslF9 Morex         TCAYEDGTEWGNDVGWVYNIATEDVVTGFRLHRTGWRSTYCAMEPDAFRGTAPINLTERL   596
CslF9_like Morex    TCAYEDGTEWGDGVGWVYDMATEDAVTGFRLHRTGWRSMYCDMEPPAFRGTAPINMTERM   513
                                        Glyco_tranf_GTA_type

ABZ01581.1          YQILRWSGGSLEMFFSRFCPLLAGRRLHPMQRVAYINMTTYPVSTFFILMYYFYPVMWLF   656
CslF9 Morex         YQILRWSGGSLEMFFSRFCPLLAGRRLHPMQRVAYINMTTYPVSTFFILMYYFYPVMWLF   656
CslF9_like Morex    YQILRWSGGSLEVFFSRFCPLLAGGRLHPMQRVAYTNMTFYPLSALFVVCYHLLPLMWVF   573
                                        Glyco_tranf_GTA_type

ABZ01581.1          QGEFYIQRPFQTFALFVVVVIATVELIGMVEIRWAGLTLLDWVRNEQFYIIGTTGVYPMA   716
CslF9 Morex         QGEFYIQRPFQTFALFVVVVIATVELIGMVEIRWAGLTLLDWVRNEQFYIIGTTGVYPMA   716
CslF9_like Morex    NGRFYIQKPYPTYVMYVLVIIVSNEVIGMVEIVWAGLTLLDWFRNEQFYMICATGVYPTA   633
                                        Glyco_tranf_GTA_type

ABZ01581.1          MLHIILRSLGIKGVSFKLTAKKLTGGARERLAELYDVQWVPLLVPTVVVMAVNVAAIGAA   776
CslF9 Morex         MLHIILRSLGIKGVSFKLTAKKLTGGARERLAELYDVQWVPLLVPTVVVMAVNVAAIGAA   776
CslF9_like Morex    VLHVVLRSLGLKGMSFKMTAKQLTTGARERFAELYDVQWAPLLIPTLVVIAVNVVAIGAA   693
                  Glyco_tranf_GTA_type

ABZ01581.1          AGKAIVGRWSAAQVAGAASGLVFNVWMLLLLYPFALGIMGHWSKRPYILFLVLVTAVAAT   836
CslF9 Morex         AGKAIVGRWSAAQVAGAASGLVFNVWMLLLLYPFALGIMGHWSKRPYILFLVLVTAVAAT   836
CslF9_like Morex    VGKAVTWGWSAGQVVEAASGLMFNVWILLMFYPFALGVIGRWGKRPYVLFAMFMAAFAAI   753

ABZ01581.1          ASVYVALAGSLLYLHSGIKLV....                                      857
CslF9 Morex         ASVYVALAGSLLYLHSGIKLV....                                      857
CslF9_like Morex    AAVYVAVLAALAGNLPYFQLVHWSV                                      778
```

Figure S11 Alignment of amino acid sequences of putativeCslF9 and CslF9-like proteins from barley cultivar Morex and the two accessions. Domains are indicated by bars and labels below the alignment. Glycosyltransferase family A (GT-A) includes diverse families of glycosyltransferases with a common GT-A type structural fold.
