# Supplementary material for: Transcriptome Assembly and Analysis of Tibetan Hulless Barley (Hordeum vulgare L. var. nudum) Developing Grains, with Emphasis on Quality Properties
Source: PLoS One. 2014 May 28;9(5):e98144. doi: 10.1371/journal.pone.0098144 (PMC4037191; doi:10.1371/journal.pone.0098144)
Supplement: Table S1 — Validation of ten differentially expressed genes using Q-PCR validation. Note: NM, Nimubai; XQ, XQ754. (DOCX) [file pone.0098144.s012.docx]

Table S1. Validation of ten differentially expressed genes using Q-PCR validation

| Gene ID | XQ_RPKM | NM_RPKM | Log2(NM_RPKM/XQ_RPKM) | Annotation | Primers |
| --- | --- | --- | --- | --- | --- |
| Unigene7327 | 39.47 | 7.24 | -2.45 | Glucan endo-1,3-beta-glucosidase, acidic isoform | 5' TGCCTCTTATTGTTTGTCTCC3'  5'TGCGGGCTTTGTACTTGA3' |
| Unigene4770 | 45.59 | 22.67 | -1.01 | ADP-glucose pyrophosphorylase small subunit | 5'ACGGTATAGGTGATCTCCAGC3'  5'CGGCTACAAGAATGACGGTT3' |
| Unigene13191 | 3222.53 | 1858.73 | -0.79 | ribulose-1,5-bisphosphate carboxylase | 5'TTACTTGAATGCGACTGCG3'  5'GCCAAACATGAATACCACCT3' |
| Unigene4315 | 95.83 | 63.96 | -0.58 | starch synthase IIa | 5'TCGTAGGCTTCCTCATAGTCCC3'  5'TCGTCGTTGCTGCTGAATG3' |
| Unigene18308 | 1356.99 | 906.45 | -0.58 | sucrose synthase | 5'AAGTTGAGCAGTGGGTAGAGG3'  5'ATTCGGGTGAATGTTAGCG 3' |
| Unigene21301 | 21.44 | 16.32 | -0.39 | zinc finger family protein | 5'CTATCCGTGTAGCCATCCC3'  5'CTAAAGCAGCGAGAACCAAG3' |
| Unigene13879 | 281.17 | 274.95 | -0.03 | 13s globulin | 5'TGAAACTCTATGGGCGTGATTG3'  5'CGACCGGACAGCAACATTCT3' |
| Unigene24180 | 234.94 | 300.80 | 0.36 | Proline-rich receptor-like protein kinase | 5'CGGGAGGGTGGTAGTAGAGC3'  5'GGCCAGATTCGAGCGAGAT3' |
| Unigene13428 | 526.08 | 1257.15 | 1.26 | avenin-like seed protein | 5'CACATCGACGGAAGGGTC3'  5'CAGCAAGGGCAGAGTTTCAC3' |
| Unigene609 | 167.86 | 848.83 | 2.34 | Ethylene receptor | 5'GACACCGCTGTTACTCTTCTACC3'  5'AGGGCCAAACTTAAACTACATAG3' |

Note: NM, Nimubai; XQ, XQ754
