# Supplementary material for: Transcriptome Assembly and Analysis of Tibetan Hulless Barley (Hordeum vulgare L. var. nudum) Developing Grains, with Emphasis on Quality Properties
Source: PLoS One. 2014 May 28;9(5):e98144. doi: 10.1371/journal.pone.0098144 (PMC4037191; doi:10.1371/journal.pone.0098144)
Supplement: Table S3 — New transcripts validated by highly homogenous ESTs of nr database. (DOCX) [file pone.0098144.s014.docx]

**Table S3.** New transcripts validated by highly homogenous ESTs of nr database.

|  | Transcripts of Morex | Accession | Query cover | Evalue | Ident |
| --- | --- | --- | --- | --- | --- |
| CSLF4-like | AK367661.1 | AK367661.1 [[1](#_ENREF_1)] | 100% | 0.0 | 100% |
| 13S-like globulin | AK376222.1 | AK376222.1[[1](#_ENREF_1)] | 100% | 0.0 | 100% |
| 13S-like globulin | AK363288 | AK360708.1[[1](#_ENREF_1)] | 100% | 0.0 | 100% |
| 11S-like globulin | MLOC_3881.1 | AK364847.1[[1](#_ENREF_1)] | 100% | 0.0 | 100% |
| 11S-like globulin | MLOC_34816.1 | AK248848.1[[1](#_ENREF_1)] | 100% | 0.0 | 99% |
| 19 kDa-like globulin | MLOC_31026.1 | AY268139.1[[2](#_ENREF_2)] | 100% | 0.0 | 100% |
